# Risk Factors of Disease Progression in IgA Nephropathy: A Systematic Review and Meta‐Analysis

**DOI:** 10.1002/iid3.70393

**Published:** 2026-02-27

**Authors:** Dan Xu, Minjie Zhang, Weiwei Liang, Lijiang Fang, Feifei Ge

**Affiliations:** ^1^ Department of Medical Laboratory Xian Yang Central Hospital Xianyang China; ^2^ Department of Medical Laboratory The Affiliated Hospital of Shaanxi University of Chinese Medicine Xianyang China; ^3^ Department of Medical Laboratory The Second Affiliated Hospital of Shaanxi University of Chinese Medicine Xianyang China; ^4^ Department of Hemodialysis Xian Yang Central Hospital Xianyang China

**Keywords:** end‐stage renal disease, IgA nephropathy, kidney disease progression, meta‐analysis, risk factors, the Oxford classification

## Abstract

**Objective:**

IgA nephropathy (IgAN) is an important cause of chronic renal failure, and nearly all patients with IgAN are at risk of developing to end‐stage renal disease (ESRD) during their lifetime. This meta‐analysis aimed to identify and evaluate risk factors associated with the progression of IgAN patients.

**Methods:**

Primary studies investigating the risk factors for predicting the progression of IgAN were included in this review. A comprehensive literature search was conducted across multiple electronic databases, including the Chinese Biological Medicine Database (CBM), China National Knowledge Infrastructure (CNKI), Cochrane Library, PubMed, Embase, Web of Science, and WANFANG, up to May 30, 2025. Two independent reviewers screened the literature based on the predefined inclusion and exclusion criteria, extracted the relevant data from the original studies, and assessed the quality of the included studies using the Newcastle‐Ottawa Scale (NOS). Meta‐analysis was performed on at least two studies reporting on a specific outcome, with pooled effect sizes expressed as hazard ratios (HRs) and 95% confidence intervals (CIs). Statistical analysis, including meta‐analysis, subgroup analysis and sensitivity analysis, was conducted using the R software. Publication bias was evaluated using Egger's test in Stata12.0.

**Results:**

A total of 53 studies, comprising 25,517 patients with IgAN, were included in the final analysis. This meta‐analysis identified 10 clinical risk factors significantly associated with IgAN progression, including mean arterial pressure (MAP) (HR = 1.02, 95% CI: 1.01–1.03), diastolic blood pressure (DBP) (HR = 1.03, 95% CI: 1.01–1.05), systolic blood pressure (SBP) (HR = 1.03, 95% CI: 1.01–1.05), serum creatinine (SCr) (HR = 1.04, 95% CI: 1.03–1.06), triglyceride (HR = 1.11, 95% CI: 1.02–1.21), 24‐h urinary protein excretion (UPE) (HR = 1.15, 95% CI: 1.12–1.18), low‐density lipoprotein cholesterol (LDL‐C) (HR = 1.37, 95% CI: 1.18–1.59), male sex (vs. female) (HR = 1.73, 95% CI: 1.16–2.59), complement C4 (C4) (HR = 1.81, 95% CI: 1.06–3.09), and hypertension (HR = 2.53, 95% CI: 1.92–3.33). The pathological features significantly predictive of IgAN progression including crescents (C1/C2) (vs. C0) (HR = 1.57, 95% CI: 1.24–1.99), C2 (vs. C0) (HR = 2.87, 95% CI: 1.65–5.01), endocapillary hypercellularity (E1) (vs. E0) (HR = 1.17, 95% CI: 1.02–1.35), segmental glomerulosclerosis (S1) (vs. S0) (HR = 2.23, 95% CI: 1.78–2.79), and tubular atrophy (T1/T2) (vs. T0) (HR = 5.12, 95% CI: 3.56–7.36), when analyzed individually, T1 (vs. T0) showed an HR = 4.59 (95% CI: 3.24–6.51), and T2 (vs. T0) showed an HR = 16.40 (95% CI: 9.65–27.87). In contrast, C1 (vs. C0) (HR = 1.41, 95% CI: 0.81–2.45) was not significantly associated with progression risk. Protective factors against lgAN progression included higher level of albumin (Alb) (HR = 0.95, 95% CI: 0.93–0.98), estimated glomerular filtration rate (eGFR) (HR = 0.96, 95% CI: 0.95–0.97), hemoglobin (Hb) (HR = 0.98, 95% CI: 0.97–0.99), complement C3 (C3) (HR = 0.97, 95% CI: 0.95–0.99). Additionally, female sex was associated with a lower risk of disease progression, with HR = 0.69 (95% CI: 0.57–0.84) and when compared directly with male HR = 0.55 (95% CI: 0.45–0.67).

**Conclusion:**

This meta‐analysis indicates that MAP, DBP, SBP, SCr, triglyceride, 24‐h UPE, LDL‐C, male sex (vs. female), C4, hypertension, C‐lesions, M‐lesions, S‐lesions, and T‐lesions in Oxford classification are significantly associated with an increased risk of IgAN progression. In contrast, higher levels of Alb, eGFR, Hb, C3, as well as female sex (both as an independent variable and compared with males), are identified as protective factors in patients with IgAN.

## 1 Introduction

IgA nephropathy (IgAN) is the most prevalence form of primary glomerulonephritis worldwide [1]. Its clinical course is highly variable, ranging from begin, asymptomatic presentations to rapid progression toward chronic kidney failure. Although a small proportion of patients experience a swift decline in renal function, a substantial develop end stage renal disease (ESRD) gradually over long‐term follow‐up [2]. Accurately identifying individuals at elevated risk of disease progression remains significant clinical challenge. Therefore, primary prevention strategies, particularly early detection and effective management of modifiable risk factors, are needed to improve renal outcomes of IgAN. IgAN diagnosis is usually performed by collecting a renal biopsy, which allows direct visualization of pathological changes in the glomeruli [3]. Histopathological remains the “gold standard” for diagnosis and prognostic evaluation in most patients. Indeed, renal biopsy findings have demonstrated substantial value in risk stratification [4]. However, renal biopsy is an invasive procedure with potential contraindications and a risk of adverse events. Furthermore, the long clinical course of IgAN often includes acute exacerbations and episodes of acute kidney injury (AKI). Under such circumstances, pathological information obtained at disease onset may not reliably predict renal progression [5]. Therefore, developing accurate and dynamic risk assessment tools for patients with IgAN holds both scientific and practical importance. Previous studies have identified several demographics, clinical and biochemical parameters associated with adverse outcomes. These include heavy proteinuria, reduced renal function, hypertension at the time of renal biopsy, the degree of renal impairment at diagnosis and laboratory measures such as serum creatinine (SCr), uric acid (UA), serum lipids, albumin (Alb), and hemoglobin (Hb) [6, 7].

In addition, the Oxford classification of IgAN, based on renal histopathology, is regarded as the most robust early histological predictor of disease progression [8]. The Oxford classification evaluates pathological features mesangial hypercellularity (M), endocapillary hypercellularity (E), segmental glomerulosclerosis (S), tubular atrophy/interstitial fibrosis (T), and crescent (C), culminating in MEST‐C scores system [6]. M0 was defined as a mesangial hypercellularity score ≤ 0.5; and M1 was defined as a mesangial hypercellularity score > 0.5 (equal to > 50% of the glomeruli with > 3 mesangial cells in the Periodic Acid‐Schiff staining). Endocapillary hypercellularity is classified E0 (absent) or E1 (present). Segmental glomerulosclerosis is scored as S0 (absent) or S1 (present, including adhesions). According to the level of tubular atrophy and interstitial fibrosis, T‐lesions were classified into three types: T0 was defined as tubular atrophy or interstitial fibrosis less than 25%, T1 was defined as > 25% and ≤ 50% tubular atrophy or interstitial fibrosis, and T2 was defined as tubular atrophy or interstitial fibrosis more than 50%, respectively. C0 is defined as the absence of crescents, C1 is defined as crescents in ≤ 25% of glomeruli, and > 25% of glomeruli as C2 [9]. Of them, the T score is the most valuable histological parameter, confirmed by a large number of original studies [10].

T‐lesions are not merely a micromorphological feature of IgAN but present a final common pathway in the progressive of many chronic kidney diseases, ultimately leading to advanced chronic kidney disease (CKD) [11]. Crescent formations are another frequently observed pathological feature in IgAN; their presence is closely associated with adverse clinicopathological characteristics, including higher proteinuria levels and elevated SCr [12]. However, the association between distinct proportions of crescents and the progression of IgAN remains a subject of ongoing debate within the medical community. The substantial clinical and pathological heterogeneity of IgAN leads to considerable inter‐individual variability in disease progression. Few studies have systematically evaluated the precise predictive value of all five Oxford classification lessons for IgAN progression. Furthermore, while some studies have identified several demographics, biochemical and pathological predictors, others have questioned their consistency and predictive reliability [13]. Therefore, a comprehensive synthesis of current evidence is urgently needed to clarify the association between these risk factors and disease progression. This meta‐analysis aims to address this gap by integrating available data to improve the understanding of risk assessment methods for IgAN progression and to inform more accurate prognostic evaluation.

## 2 Materials and Methods

### 2.1 Search Strategy

A comprehensive literature search was conducted across the Chinese Biological Medicine Database (CBM), China National Knowledge Infrastructure (CNKI), Cochrane Library, Medline via PubMed, Embase, Web of Science, and WANFANG databases. The search included original articles published between January 1, 2000 and May 30, 2025 and was restricted to studies published in English or Chinese. The following keywords or phrases were used: “IgA nephropathy” OR “glomerulonephritides” OR “IgA glomerulonephritis” OR “Immunoglobulin A nephropathy” OR “IgA type nephritis” OR “IgA nephropathy 1” combined with “kidney disease progression” OR “end‐stage renal disease” OR “kidney failure” OR “IgAN progression” OR “risk factors” OR “adverse kidney outcomes” OR “renal outcomes” OR “adverse outcomes” OR “progressive kidney disease” OR “renal disease progression” OR “deterioration of renal function” OR “renal endpoint” OR “progression to ESRD” OR “composite endpoint” OR “poor renal outcomes” OR “composite event.” Two reviewers independently screened titles and abstracts for relevance, followed by full text assessment of potentially eligible studies. Discrepancies were resolved through discussion or consultation with a third reviewer. Studies examining the association between various risk factors and IgAN progression were included. Additional relevant articles were obtained through a manual search of reference lists from retrieved studies.

### 2.2 Inclusion and Exclusion Criteria

Studies were eligible for inclusion if they met all of the following criteria: (1) Included participants with biopsy‐confirmed IgAN. (2) Reported kidney related endpoints as the primary or secondary outcome. (3) Provide data on at least one prespecified kidney outcomes. (4) Followed participants for a minimum duration of 6 months. (5) Reported effect estimates, hazard ratios (HRs) with corresponding 95% confidence intervals (CIs). (6) Analyzed at least one potential risk factor for IgAN progression.

Studies were excluded if they met any of the following conditions: (1)Included patients with secondary IgAN, such as those associated with Henoch‐Schonlein purpura, ankylosing spondylitis, psoriasis or liver disease. (2) Included patients with a second coexisting disease on kidney disease confirmed by biopsy, like diabetic nephropathy. (3) Enrolling patients who had received corticosteroids or other immunosuppressive therapy before renal biopsy. (4) Review article, case report, or conference articles.

### 2.3 Data Extraction and Quality Assessment

The literature search, data extraction, and quality assessment were conducted independently by two reviewers. The following information was extracted from each study: first author's name, year of publication, study design, primary outcome, description of the outcome events, sample size, average age, female/male (F/M) ratio, and follow‐up durations. The methodological quality of the included studies was assessed using the Newcastle‐Ottawa scale (NOS), with scores ranging from 0 to 8. Studies scoring ≥ 7 were considered high‐quality, whereas those scoring < 6 were deemed to have a high risk of bias. Two independent reviewers evaluated the quality of each study, discrepancies in study selection, or quality scoring were resolved through discussion with a third reviewer. The results of the quality assessment are shown in Table S1.

### 2.4 Statistical Analysis

All statistical analysis was conducted using R software. HRs with 95% CIs were extracted from each eligible study by two independent researchers. For both categorical and continuous variables, pooled effect sizes were calculated and reported as HRs with corresponding 95% CIs. The potential heterogeneity of the studies was assessed by the inconsistency index (*Ι* ^2^) and Cochran‐Q statistic. And *I* ^2^ values 50% ≤ were considered as indicative of low heterogeneity, whereas values > 50% indicated high heterogeneity. Statistical significance for heterogeneity was defined as *p* < 0.05 for the Cochrane Q test. In case of significant heterogeneity, a random effects model was applied for data synthesis, otherwise, a fixed‐effect model was used. To further investigate the potential source of heterogeneity, subgroup analysis was performed based on predefined covariates. Additionally, sensitivity analysis was also conducted to assess the impact of each individual study on the overall results. Publication bias was evaluated using Egger's tests, with *p* values < 0.05 indicating significant bias.

## 3 Results

### 3.1 Study Characteristics

**Figure 1:**
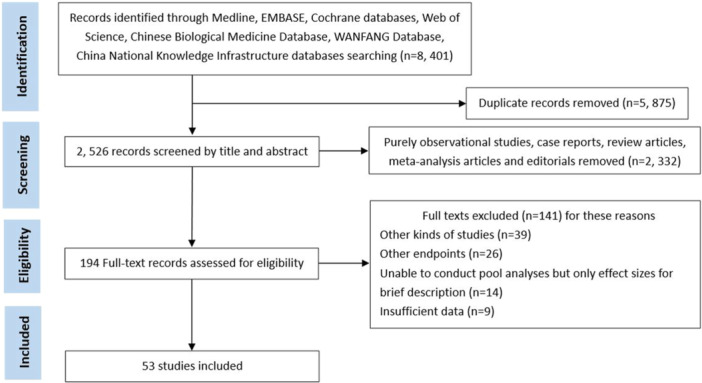
Flow diagram of the literature selection and screening process.

The initial database search yielded 8401 articles, of which 5875 articles were duplicates. After duplicates removal 2526 records remained for screening. Following full‐text assessment, 141 articles were excluded and 53 articles [14, 15, 16, 17, 18, 19, 20, 21, 22, 23, 24, 25, 26, 27, 28, 29, 30, 31, 32, 33, 34, 35, 36, 37, 38, 39, 40, 41, 42, 43, 44, 45, 46, 47, 48, 49, 50, 51, 52, 53, 54, 55, 56, 57, 58, 59, 60, 61, 62, 63, 64, 65, 66] involving 25,517 patients were included in the final analysis. The sample sizes ranged from 24 to 1818 patients. Across studies, the percentage of males’ patients was 50.47%, with a mean follow‐up duration ranging from 6 to 113 months; three studies [31, 32, 56] did not report follow‐up data. The follow‐up endpoints varied among the included studies. The study included 43 retrospective cohort studies, 8 prospective cohort studies, and 2 observational cohort studies. The detailed characteristics of the included articles are shown in Table S2, and the article selection and screening processes are illustrated in Figure 1.

### 3.2 Data Synthesis and Meta‐Analysis

**Figure 2:**
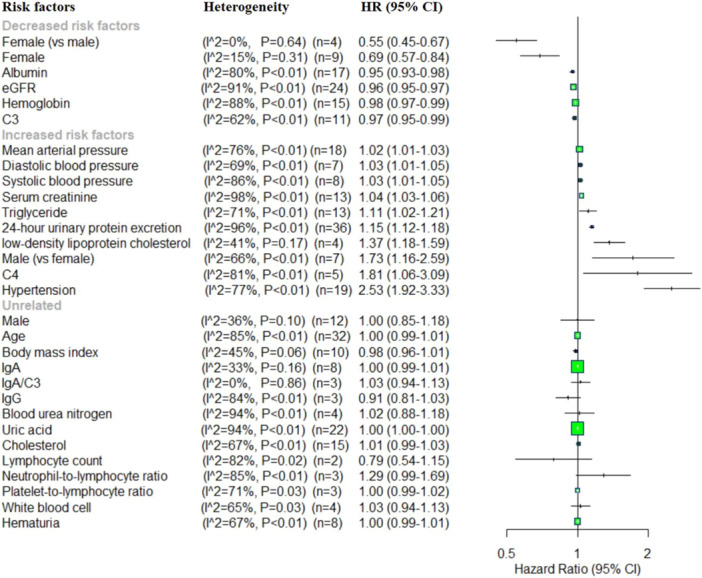
Forest plot of the pooled HRs for demographics and clinical risk factors in IgAN progression.

Demographics and clinical data: this meta‐analysis showed that the identified several factors significantly associated with an increased risk of IgAN progression. These included mean arterial pressure (MAP) (HR = 1.02, 95% CI: 1.01–1.03), diastolic blood pressure (DBP) (HR = 1.03, 95% CI: 1.01–1.05), systolic blood pressure (SBP) (HR = 1.03, 95% CI: 1.01–1.05), SCr (HR = 1.04, 95% CI: 1.03–1.06), triglyceride (HR = 1.11, 95% CI: 1.02–1.21), 24‐h urinary protein excretion (UPE) (HR = 1.15, 95% CI: 1.12–1.18), low‐density lipoprotein cholesterol (LDL‐C) (HR = 1.37, 95% CI: 1.18–1.59), male sex (vs. female) (HR = 1.73, 95% CI: 1.16–2.59), complement C4 (C4) (HR = 1.81, 95% CI: 1.06–3.09), and hypertension (HR = 2.53, 95% CI: 1.92–3.33). Conversely, several variable factors were associated with a reduce risk progression, including female sex (vs. male) (HR = 0.55, 95% CI: 0.45–0.67), and HR = 0.69, 95% CI: 0.57–0.84 in sperate analyses, serum Alb (HR = 0.95, 95% CI: 0.93–0.98), estimated glomerular filtration rate (eGFR) (HR = 0.96, 95% CI: 0.95–0.97), Hb (HR = 0.98, 95% CI: 0.97–0.99) and complement (C3) (HR = 0.97, 95% CI: 0.95–0.99). No statistically significant associations were observed for male (HR = 1.00, 95% CI: 0.85–1.18), age (HR = 1.00, 95% CI: 0.99–1.01), body mass index (BMI) (HR = 0.98, 95% CI: 0.96–1.01), IgA (HR = 1.00, 95% CI: 0.99–1.01), IgA/C3 ratio (HR = 1.03, 95% CI: 0.94–1.13), IgG (HR = 0.91, 95% CI: 0.81–1.03), blood urea nitrogen (BUN) (HR = 1.02, 95% CI: 0.88–1.18), UA (HR = 1.00, 95% CI: 1.00–1.00), cholesterol (HR = 1.01, 95% CI: 0.99–1.03), lymphocyte (LY) count (HR = 0.79, 95% CI: 0.54–1.15), neutrophil‐to‐lymphocyte ratio (NLR) (HR = 1.29, 95% CI: 0.99–1.69), platelet‐to‐lymphocyte ratio (PLR) (HR = 1.00, 95% CI: 0.99–1.02), white blood cells (WBCs) (HR = 1.03, 95% CI: 0.94–1.13), and hematuria (HR = 1.00, 95% CI: 0.99–1.01) (Figure 2).

**Figure 3:**
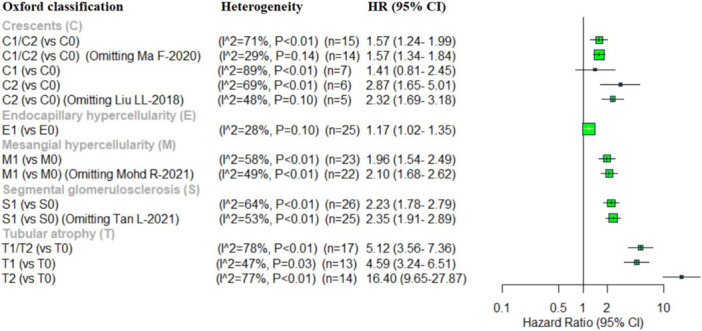
Forest plot of the pooled HRs for five pathologic lesions and sensitivity analysis in IgAN progression.

Oxford classification: regarding the correlation between five pathologic lesions of Oxford classification and kidney disease progression in IgAN, this meta‐analysis identified a significant correlation between E, M, S and T scores. The pooled results showed that C1/C2 (vs. C0) (HR = 1.57, 95% CI: 1.24–1.99), C2 (vs. C0) (HR = 2.87, 95% CI: 1.65–5.01), E1 (vs. E0) (HR = 1.17, 95% CI: 1.02–1.35), M1 (vs. M0) (HR = 1.96, 95% CI: 1.54–2.49), S1 (vs. S0) (HR = 2.23, 95% CI: 1.78–2.79), T1/T2 (vs. T0) (HR = 5.12, 95% CI: 3.56–7.36), T1 (vs. T0) (HR = 4.59, 95% CI: 3.24–6.51) and T2 (vs. T0) (HR = 16.40, 95% CI: 9.65–27.87) were significantly associated with increased risk of IgAN progression. In contrast, C1 (vs. C0) (HR = 1.41, 95% CI: 0.81–2.45) showed a positive association with kidney disease progression in IgAN, however, this correlation did not reach statistical significance (Figure 3).

### 3.3 Heterogeneity Test and Sensitivity Analysis

**Figure 4:**
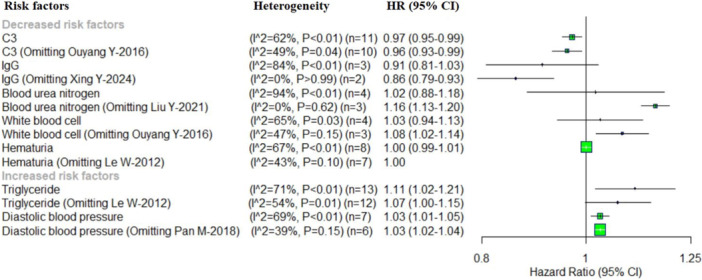
Forest plot of the sensitivity analysis for demographics and clinical risk factors.

A heterogeneity analysis was conducted for the identified risk factors, revealing significant heterogeneity for several variables, including Alb (*I* ^2^ = 80%, *p* < 0.01), eGFR (*I* ^2^ = 91%, *p* < 0.01), Hb (*I* ^2^ = 88%, *p* < 0.01), C3 (*I* ^2^ = 62%, *p* < 0.01), MAP (*I* ^2^ = 76%, *p* < 0.01), DBP (*I* ^2^ = 69%, *p* < 0.01), SBP (*I* ^2^ = 86%, *p* < 0.01), SCr (*I* ^2^ = 98%, *p* < 0.01), triglyceride (*I* ^2^ = 71%, *p* < 0.01), 24‐h UPE (*I* ^2^ = 96%, *p* < 0.01), male (vs. female) (*I* ^2^ = 66%, *p* < 0.01), C4 (*I* ^2^ = 81%, *p* < 0.01), hypertension (*I* ^2^ = 77%, *p* < 0.01), age (*I* ^2^ = 85%, *p* < 0.01), IgG (*I* ^2^ = 84%, *p* < 0.01), BUN (*I* ^2^ = 94%, *p* < 0.01), UA (*I* ^2^ = 94%, *p* < 0.01), cholesterol (*I* ^2^ = 67%, *p* < 0.01), LY count (*I* ^2^ = 82%, *p* < 0.01), NLR (*I* ^2^ = 85%, *p* < 0.01), PLR (*I* ^2^ = 71%, *p* = 0.03), WBCs (*I* ^2^ = 65%, *p* = 0.03), and hematuria (*I* ^2^ = 67%, *p* < 0.01). Sensitivity analysis, conducted using R software, involved iteratively omitting individual studies to determine potential sources of heterogeneity. Excluding “Ouyang 2016” reduced heterogeneity for C3 (HR = 0.96, 95% CI, 0.93–0.99; *I* ^2^ = 49%, *p* = 0.04) while removing “Le W 2012” reduced heterogeneity for triglyceride (HR = 1.07, 95% CI, 1.00–1.15; *I* ^2^ = 54%, *p* = 0.01). Excluding “Xing Y 2024” completely eliminated heterogeneity for IgG (HR = 0.86, 95% CI, 0.79–0.93; *I* ^2^ = 0%, *p* > 0.99). Similarly, when “Liu Y 2021” was removed, heterogeneity for BUN was eliminated (HR = 1.16, 95% CI, 1.13–1.20; *I* ^2^ = 0%, *p* = 0.62). Omitting “Ouyang 2016” also eliminated significant heterogeneity for WBCs (HR = 1.08, 95% CI, 1.02–1.14; *I* ^2^ = 47%, *p* = 0.15) and excluding “Le W 2012” eliminated heterogeneity for hematuria (HR = 1.00; *I* ^2^ = 43%, *p* = 0.10). When “Pan M 2018” was removed, no significant heterogeneity was observed for DBP (HR = 1.03, 95% CI, 1.02–1.04; *I* ^2^ = 39%, *p* = 0.15). No single study significantly affected the observed association of these risk factors with the progression of IgAN (C3, hematuria and DBP). However, the pooled HR of the IgG became significantly associated with decreased risk of IgAN progression when the study by “Xing Y 2024” was excluded. In contrast, the omission of “Liu Y 2021” significantly affected the pooled HR for BUN, while the omission of “Ouyang 2016” had a substantial impact on the pooled HR for WBCs. Excluding either “Liu Y 2021” or “Ouyang 2016” shifted the pooled HR from a statistically non‐significant to significant association. Meanwhile, omitting a single study, no significant heterogeneity was observed for IgG, BUN, WBCs, hematuria, and DBP (Figure 4).

Additionally, we investigated heterogeneity among the five pathologic lesions in Oxford classification. Significantly high heterogeneity was observed for C1/C2(vs. C0), C1 (vs. C0), C2 (vs. C0), M1 (vs. M0), S1 (vs. S0), S1, T1/T2 (vs. T0), T1 (vs. T0), and T2 (vs. T0). Sensitivity analysis was conducted to identify possible sources of heterogeneity for each score. For C1/C2(vs. C0), heterogeneity was reduced to a non‐significant level (HR = 1.57, 95% CI, 1.34–1.84; *I* ^2^ = 29%, *p* = 0.14) upon exclusion of “Ma F 2020.” Similarly, for C2 (vs. C0) exclusion of “Liu LL 2018” reduced heterogeneity to a non‐significant level (HR = 2.32, 95% CI, 1.69–3.18; *I* ^2^ = 48%, *p* = 0.10). Omitting “Mohd R 2021” decreased heterogeneity for M1 (vs. M0) (HR = 2.10, 95% CI, 1.68–2.62; *I* ^2^ = 49%, *p* < 0.01), while removal of Tan L 2021” reduced heterogeneity for S1 (vs. S0) (HR = 2.35, 95% CI, 1.91–2.89; *I* ^2^ = 53%, *p* < 0.01) (Figure 3).

### 3.4 Subgroup Analysis

**Figure 5:**
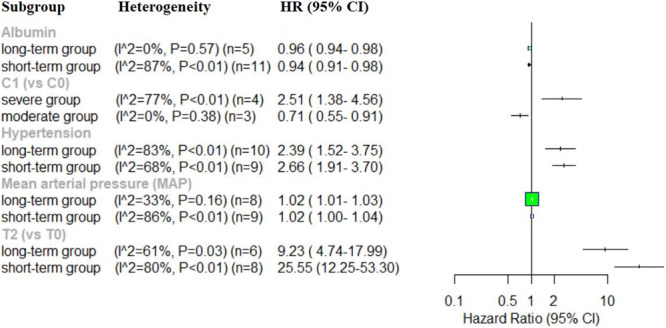
Forest plot of subgroup analysis.

Excluding any single study did not significantly reduce the heterogeneity across the studies in the analysis of Alb, eGFR, Hb, MAP, SBP, SCr, 24‐h UPE, male (vs. female), C4, hypertension, age, UA, cholesterol, LY count, NLR, and PLR. A subgroup analysis was conducted based on follow‐up period and primary outcome, the follow‐up period was categorized as “short‐term” (< 47.8 months) and “long‐term” (≥ 47.8 months), while the primary outcomes were classified into the “severe” group and “moderate” groups, to identify the potential sources of heterogeneity among the studies of the aforementioned risk factors. In the subgroup analysis of Alb, the “short‐term” follow‐up group exhibited higher heterogeneity (*I* ^2^ = 87%, *p* < 0.01) compared to the “long‐term” follow‐up group (*I* ^2^ = 0%, *p* = 0.57), with no significant heterogeneity observed in the “long‐term” subgroup. Similarly, for MAP, the “short‐term” follow‐up group exhibited higher heterogeneity (*I* ^2^ = 86%, *p* < 0.01) than the “long‐term” group (*I* ^2^ = 33%, *p* = 0.16), with no significant heterogeneity in the “long‐term” subgroup. For hypertension, the “short‐term” group exhibited reduced heterogeneity (*I* ^2^ = 68%, *p* < 0.01) compared to the “long‐term” group (*I* ^2^ = 83%, *p* < 0.01). However, T2 (vs. T0), the “short‐term” follow‐up group had higher heterogeneity (*I* ^2^ = 80%, *p* < 0.01) compared to the “long‐term” group (*I* ^2^ = 61%, *p* = 0.03). Subgroup analysis based on primary outcome revealed that the “severe” group exhibited higher heterogeneity (*I* ^2^ = 77%, *p* < 0.01) than the “moderate” group (*I* ^2^ = 0%, *p* = 0.38), with no significant heterogeneity observed in the “moderate” group for C1 (vs. C0) (Figure 5). Both sensitivity and subgroup analysis were performed to identify potential sources of heterogeneity, however, no contributing factors were identified for the eGFR, Hb, SBP, SCr, 24‐h UPE, male (vs. female), C4, age, UA, cholesterol, LY count, NLR, PLR, and T1/T2 (vs. T0).

### 3.5 Risk of Bias Assessment

Publication bias in the included studies was assessed. Egger's tests revealed no significant publication bias, except for the analysis of SBP, SCr, triglyceride, 24‐h UPE, LDL‐C, UA, and C1/C2 (vs. C0) (as shown in Table 1).

**Table 1:** Risk factors associated with the progression of IgAN.

Factors	Pooled effect		Heterogeneity		Egger's test	
	HR [95%CI]	*p*	*I* ^2^ (%)	*p*	*t*	*p*
Decreased risk factors						
Female (vs. male)	0.55 (0.45–0.67)	< 0.01	0	0.64	−0.09	0.936
Female	0.69 (0.57–0.84)	< 0.01	15	0.31	2.31	0.054
Albumin	0.95 (0.93–0.98)	< 0.01	80	< 0.01	−1.08	0.296
eGFR	0.96 (0.95–0.97)	< 0.01	91	< 0.01	0.42	0.680
Hemoglobin	0.98 (0.97–0.99)	< 0.01	88	< 0.01	−1.32	0.209
C3	0.97 (0.95–0.99)	0.01	62	< 0.01	−0.07	0.948
C3 (Omitting Ouyang Y‐2016)	0.96 (0.93–0.99)	0.01	49	0.04	0.82	0.435
Increased risk factors						
Mean arterial pressure	1.02 (1.01–1.03)	< 0.01	76	< 0.01	1.29	0.214
Diastolic blood pressure	1.03 (1.01–1.05)	< 0.01	69	< 0.01	0.76	0.483
Diastolic blood pressure (Omitting Pan M‐2018)	1.03 (1.02–1.04)	< 0.01	39	0.15	−0.81	0.462
Systolic blood pressure	1.03 (1.01−1.05)	< 0.01	86	< 0.01	3.38	0.015^a^
Serum creatinine	1.04 (1.03−1.06)	< 0.01	98	< 0.01	2.59	0.027^a^
Triglyceride	1.11 (1.02−1.21)	0.02	71	< 0.01	2.72	0.020^a^
Triglyceride (Omitting Le W‐2012)	1.07 (1.00−1.15)	0.06	54	0.01	2.44	0.035^a^
24‐h urinary protein excretion	1.15 (1.12−1.18)	< 0.01	96	< 0.01	5.84	0.000^a^
Low‐density lipoprotein cholesterol	1.37 (1.18−1.59)	< 0.01	41	0.17	5.42	0.032^a^
Male (vs. female)	1.73 (1.16−2.59)	< 0.01	66	< 0.01	0.20	0.851
C4	1.81 (1.06−3.09)	0.03	81	< 0.01	0.60	0.590
Hypertension	2.53 (1.92−3.32)	< 0.01	77	< 0.01	−0.72	0.484
Unrelated						
Male	1.00 (0.85–1.18)	0.99	36	0.10	0.22	0.829
Age	1.00 (0.99–1.01)	0.58	85	< 0.01	−0.13	0.897
Body mass index	0.98 (0.96–1.01)	0.20	45	0.06	−0.61	0.559
IgA	1.00 (0.99–1.01)	0.06	33	0.16	−1.57	0.167
IgA/C3	1.03 (0.94–1.13)	0.52	0	0.86	−0.06	0.962
IgG	0.91 (0.81–1.03)	0.15	84	< 0.01	−5.46	0.115
IgG (Omitting Xing Y‐2024)	0.86 (0.79–0.93)	< 0.01	0	> 0.99	NA	NA
Blood urea nitrogen	1.02 (0.88–1.18)	0.83	94	< 0.01	−1.76	0.221
Blood urea nitrogen (Omitting Liu YY‐2021)	1.16 (1.13–1.20)	< 0.01	0	0.62	4.59	0.136
Uric acid	1.00 (1.00–1.00)	0.03	94	< 0.01	3.29	0.004^a^
Cholesterol	1.01 (0.99–1.03)	0.37	67	< 0.01	1.58	0.139
Lymphocyte count	0.79 (0.54–1.15)	0.21	82	0.02	NA	NA
Neutrophil‐to‐lymphocyte ratio	1.29 (0.99–1.69)	0.06	85	< 0.01	0.80	0.571
Platelet‐to‐lymphocyte ratio	1.00 (0.99–1.02)	0.57	71	0.03	3.31	0.187
White blood cell	1.03 (0.94–1.13)	0.48	65	0.03	−0.81	0.503
White blood cell (Omitting Ouyang Y‐2016)	1.08 (1.02–1.14)	< 0.01	47	0.15	−0.73	0.597
Hematuria	1.00 (0.99–1.01)	0.89	67	< 0.01	−1.02	0.348
Hematuria (Omitting Le W‐2012)	1.00 (1.00–1.00)	0.97	43	0.10	−0.39	0.712
Oxford classification						
C1/C2 (vs. C0)	1.57 (1.24–1.99)	< 0.01	71	< 0.01	5.73	0.000^a^
C1/C2 (vs. C0) (Omitting Ma F‐2020)	1.57 (1.34–1.84)	< 0.01	29	0.14	2.04	0.064
C1 (vs. C0)	1.41 (0.81–2.45)	0.22	89	< 0.01	1.02	0.353
C2 (vs. C0)	2.87 (1.65–5.01)	< 0.01	69	< 0.01	0.18	0.863
C2 (vs. C0) (Omitting Liu LL‐2018)	2.32 (1.69–3.18)	< 0.01	48	0.10	0.08	0.943
E1 (vs. E0)	1.17 (1.02–1.35)	0.03	28	0.10	1.18	0.250
S1 (vs. S0)	2.23 (1.78–2.79)	< 0.01	64	< 0.01	−0.18	0.857
S1 (vs. S0) (Omitting Tan L‐2021)	2.35 (1.91–2.89)	< 0.01	53	< 0.01	−1.50	0.147
T1/T2 (vs. T0)	5.12 (3.56–7.36)	< 0.01	78	< 0.01	0.85	0.407
T1 (vs. T0)	4.59 (3.24–6.51)	< 0.01	47	0.03	2.16	0.054
T2 (vs. T0)	16.40 (9.65–27.87)	< 0.01	77	< 0.01	1.08	0.300
M1 (vs. M0)	1.96 (1.54–2.49)	< 0.01	58	< 0.01	−1.57	0.131
M1 (vs. M0) (Omitting Mohd R‐2021)	2.10 (1.68–2.62)	< 0.01	49	< 0.01	−1.63	0.120

^a^ *p* < 0.05, significant publication bias was observed.

## 4 Discussion

IgAN is a common, multifactorial kidney disease influenced by a complex interplay of genetic, immunological, and environmental factors. Autoimmunity and inflammation are considered the fundamental mechanisms driving its development and progression [67]. Over recent decades, numerous factors have been identified as being associated with the progression of IgAN. Although clinical and histopathological risk factors have classically been applied in routine practice to guide the therapeutic plan for IgAN [68], the predictive role of some of the identified risk factors remains under investigation. In this meta‐analysis, we comprehensively identify the association between various clinical factors and the progression of IgAN. Additionally, the Oxford classification is widely accepted as a histopathology tool for predicting renal outcomes in IgA nephropathy [6]. Previous studies showed that the presence and severity of pathological changes defined by the MEST‐C scoring in the Oxford classification are associated with differing risks of disease progression [69]. Accordingly, this analysis explored the correlation between the five pathological lesions in the Oxford classification and the advancement of kidney disease in IgAN. Our finding indicates that, in the blood pressure category, higher MAP, higher SBP, higher DBP and the presence of hypertension were all significantly associated with an increased risk of IgAN progression. Moreover, IgAN itself can contribute to the onset of hypertension, given the kidney's central role in the regulation of blood pressure. In IgAN, the deposition of immune complexes in the mesangial areas of the glomeruli triggers local inflammation and subsequent kidney damage, thereby exacerbating hypertension and disease progression [70, 71]. Kidney damage leads to sodium retention and activation of the renin‐angiotensin‐aldosterone system, resulting in renal hypertension. Therefore, effective blood pressure control is essential for disease management and has a significant influence on quality of life [72]. Intensive blood pressure control strategies, aimed at reducing cardiovascular risks by targeting SBP levels below conventional thresholds, have emerged as a pivotal approach in hypertension management [72, 73]. However, no studies have yet reported on the relationship between effective blood pressure control and kidney prognosis in patients with IgAN. Among biochemical parameters, this meta‐analysis demonstrated that elevated triglycerides and LDL‐C levels were associated with increased risk of IgAN progression, whereas cholesterol levels were not significantly associated. IgAN is often accompanied by abnormal lipid metabolism [74], which is a known risk factor for cardiovascular events and can influence disease progression and prognosis. Hyperlipidemia, one of the most common types of dyslipidemia in IgAN, can cause glomerular sclerosis and renal tubular fibrosis, thereby increasing the risk of renal dysfunction progression [75]. The podocyte apoptosis and endothelial dysfunction induced by hypertriglyceridemia are possible underlying mechanism [76]. In addition, studies have shown that lipid accumulation and lipotoxicity can impair the function of glomerular podocytes and proximal renal tubular epithelial cells [77]. Both hypercholesterolemia and hypertriglyceridemia have been reported to be associated with deterioration of renal function in adult patients with IgAN [21].

Proteinuria is an established independent predictor for IgAN progression and plays an important role in both the diagnosis of kidney disease and the monitoring of disease activity [78]. It is the second most common clinical feature of IgAN after hematuria, and the most widely used method for its assessment is 24‐h UPE. Previous research [79] has shown that early remission of proteinuria, especially spontaneous remission within a short period, can significantly improve the prognosis of IgAN patients with nephrotic syndrome (NS). This underscores the importance of considering both the clinical significance of proteinuria and its impact on disease progression. In our study, the 24‐h UPE at the time of biopsy was found to be associated with kidney disease progression in IgAN patients. Additionally, we observed that elevated SCr, higher C4 levels, and male sex (vs. female) were associated with IgAN progression. In recent years, studies demonstrated that complement activation occurred locally in IgAN, several studies reported that systemic complement activation may also be present. In particular, Tringali et al. showed that decreased C3 and increased C4 levels are associated with poor renal prognosis in IgAN [80], suggesting that complement system activation is crucial in IgAN pathogenesis. Pan et al. showed that increased serum C4 and decreased C3 levels at renal biopsy were associated with poor renal prognosis in patients with IgAN [81]. Moreover, decreased serum C3 levels were independently predictive of renal outcome in the multivariate analysis [20]. This study also showed that serum C3 levels at the time of renal biopsy were significantly associated with decreased risks of IgAN progression. However, serum C4 levels were identified as an independent risk factor for disease progression. The mechanism underlying the association between elevated serum C4 levels and poor IgAN prognosis remains unclear. The present study demonstrated that high serum C4 levels correlated with more severe clinical and pathological manifestations [15], which were in turn strongly associated with increased risk of renal progression [82].

Elevated SCr at the time of IgAN diagnosis generally reflects impaired renal function and more advanced disease severity. SCr serves as an indirect marker of eGFR and may therefore reflect the extent of underlying structural and functional renal damage. Numerous studies have shown that progression of renal injury is significantly associated with elevated baseline SCr levels. These findings suggest that increased SCr may result from glomerular injury and declining filtration capacity, which are central features of lgAN progression. Accordingly, elevated SCr at diagnosis can be interpreted as an important prognostic indicator and a risk factor for accelerated progression to end‐stage renal disease ESRD. This study suggests that a high SCr level is a risk factor for the progression of IgAN. Intriguingly, serum UA is also attracting increasing attention as a potential risk factor. However, previous studies exploring the relationship between serum UA levels and IgAN progression have yielded inconsistent results. Some studies found that hyperuricemia may be an independent risk factor for the development of ESRD in patients with IgAN [83, 84], whereas other studies found that the association was not statistically significant. It is important to note that the treatment and management of IgAN is a long‐term process, and serum UA levels may fluctuate as the disease progresses. In our study, serum UA levels and BUN levels were not significantly associated with IgAN progression. As for laboratory indicators, a complete blood count test, commonly performed in clinical practice, measures key blood components such as WBCs, neutrophils (NEs), lymphocytes (LYs), and platelets (PLTs). In our study, we evaluated the effects of LY count, NLR, PLR, and WBC count on predicting IgAN progression. The results showed that none of the hematological indices were associated with IgAN progression suggesting that the factors beyond hematological parameters may play a more substantial role in disease advancement.

Additionally, we found that the presence of hematuria at the time of biopsy was not associated with kidney disease progression in IgAN patients. Some studies have revealed that remission of hematuria may delay the progression of renal function and reduce the occurrence of adverse renal outcomes [85]. It is important to note that conflicting results among different studies highlight the complexity of IgAN and underscore the need for more clinical evidence to clarify its guiding significance for clinical practice. We also found that IgA, IgA/C3, IgG, sex (male), age, and BMI at diagnosis were not associated with IgAN progression. Furthermore, previous meta‐analysis indicated that high BMI was significantly associated with the incidence of adverse renal outcomes and deteriorated eGFR at the last follow‐up [86]. Iseki et al. presented that obesity was associated with increased risk for the incidence of ESRD in CKD [87]. However, in this study, based on meta‐analysis of 10 studies, we found no significant correlation between BMI and IgAN progression. Anemia is a common complication of CKD, some studies have investigated the effect of serum Hb on the renal progression of IgAN. Moreover, Oh et al. have suggested that hemoglobin at diagnosis was an independent predictor for IgAN progression [88]. Renal anemia is due to various mechanisms, previous studies emphasize the role of chronic hypoxia in the tubulointerstitium as a final common pathway to ESRD. Anemia might accelerate renal function decline by inducing tubulointerstitial hypoxia [89]. This meta‐analysis demonstrated that increased Hb levels at the time of renal biopsy were significantly associated with a decreased risk of IgAN progression. Therefore, to determine whether early clinical intervention for anemia might delay IgAN progression, high‐quality RCTs are needed. In healthy men and women, an approximately 12% difference of mean Hb levels in venous blood has been reported, with men having higher Hb levels than women [90]. Based on our findings, increased Hb level was identified as a protective factor in IgAN patients. However, we found that male sex was associated with a higher risk of the progression of IgAN compared with female sex. Despite the difference in venous Hb levels, there was no interaction effect between Hb and sex on the progression of IgAN. We presumed that although increased Hb is significantly associated with a decreased risk of IgAN progression, it remains unknown whether early clinical intervention for renal anemia has renoprotective effects.

The Oxford classification is widely accepted as a histopathology tool for predicting kidney outcomes in IgAN. M‐lesions, S‐lesions, particularly T1 and T2, and C‐lesions are strongly associated with disease progression, independent of laboratory and clinical parameters. The predictive role of E‐lesions has been linked to immunosuppressive therapy [13, 91]. Park et al. reported that M‐lesions, S‐lesions, and C‐lesions, which reflect the intraglomerular activity of the disease, were significant risk factors for poor prognosis [92]. In our study, the association of C1/C2 (vs. C0) with IgAN progression was significant, whereas significant high heterogeneity was observed. Sensitivity analysis indicated that “Ma F 2020” primarily contributed to this heterogeneity in the analysis of C1/C2 (vs. C0) (*I* ^2^ = 29%, *p* = 0.14, omitting “Ma F 2020”). We also found a significant association between C2 and IgAN progression, whereas no association was identified for C1 and IgAN progression. Removing “Liu LL 2018” reduced heterogeneity in the C2 (vs. C0) analysis (*I* ^2^ = 48%, *p* = 0.10, omitting “Liu LL 2018”). E1 (vs. E0) was not associated with IgAN progression, and sensitivity analysis revealed that this association of E1 (vs. E0) with IgAN progression was unstable and altered by the exclusion of individual studies. Although Chakera et al. found that the baseline eGFR, proteinuria, and E score were independent predictors of time to ESRD and rapid decline in eGFR [93]. E‐lesions typically not a significant prognostic factor in native IgAN have been associated with poor graft outcomes in recurrent disease. Overall, the evidence supporting E‐lesions as a risk factor for IgAN progression is marginal. Lim et al. reported that adding clinical variables at the time of biopsy (elevated SCr level, proteinuria, and decreased eGFR) to S‐lesions and T‐lesions improved the prediction of IgAN progression [94]. Our meta‐analysis produced similar results, with T‐lesions showing the highest risk association: HR increased from 4.59 in T1 (vs. T0) to 5.12 and 16.40 in the T1/T2 (vs. T0) and T2 (vs. T0), indicating that tubular and interstitial lesions are strong predictors of kidney disease outcomes. The S‐lesions are also well recognized for their predictive value in IgAN [95, 96], and prior studies using repeated eGFR measurement have shown steeper declines in patients with S‐lesions than in those without [95, 97]. Our findings confirm that C‐lesions, M‐lesions, S‐lesions, and T‐lesions (but not E‐lesions and C1) in the Oxford classification are associated strongly with IgAN progression. While the Oxford classification is a useful predictor, each MEST‐C component can be influenced by several confounders, and the total MEST‐C score may provide a more comprehensive measure of disease severity, potentially reconciling differences among individual studies. Treatment decisions for IgAN generally rely on both clinical and histological findings, and the renal prognosis is predicted based on these data. Together, these classifications support more accurate decision‐making by nephrologists, emphasizing that both histological and clinical factors are essential for optimal IgAN management.

Sensitivity analysis was performed using R software to identify possible sources of heterogeneity among the included studies, which examined 23 demographics and clinical factors as well as the five pathologic lesions defined by the Oxford classification. Omitting a single study did not significantly affect the pooled HR for Alb, eGFR, Hb, MAP, DBP, SBP, SCr, 24‐h UPE, male (vs. female), C4, mesangial, hypertension, age, UA, cholesterol, LY count, NLR, PLR, hematuria, C‐lesions, M‐lesions, S‐lesions, and T‐lesions. However, the pooled HR for C3, IgG, BUN, WBCs, triglyceride and E‐lesions were significantly affected by the removal of a single study. Additionally, no significant heterogeneity was observed for WBCs, hematuria, DBP, C1/C2 (vs. C0) and C1 (vs. C0); heterogeneity was eliminated for IgG and BUN; and heterogeneity for C3, triglyceride, M1 (vs. M0) and S1 (vs. S0) was reduced an omitting individual study. Sensitivity analysis revealed that the heterogeneity of WBCs, hematuria, DBP, C1/C2 (vs. C0), C1 (vs. C0), IgG, BUN, C3, triglyceride, M1 (vs. M0) and S1 (vs. S0) was primarily driven by a single study. Due to the pooled HRs for C3, IgG, BUN, WBCs, triglyceride, E‐lesions were significantly affected by the omission of a single study and the heterogeneity results for these variables were unstable, the reliability of the evidence from this meta‐analysis for these factors remains uncertain.

Subgroup analysis was conducted to identify the source of heterogeneity for Alb, eGFR, Hb, MAP, SBP, SCr, 24‐h UPE, male (vs. female), C4, hypertension, age, UA, cholesterol, LY count, NLR, and PLR. However, these factors did not explain the unobserved heterogeneity. In the analysis of Alb (*I* ^2^ = 80%, *p* < 0.01), heterogeneity was eliminated in the “long‐term” follow‐up group (*I* ^2^ = 0%, *p* = 0.57) compared to the “short‐term” follow‐up group (*I* ^2^ = 87%, *p* < 0.01). Similarly, in the analysis of MAP (*I* ^2^ = 76%, *p* < 0.01), no significant heterogeneity was observed in the “long‐term” follow‐up group (*I* ^2^ = 33%, *p* = 0.16) compared to the “short‐term” follow‐up group (*I* ^2^ = 86%, *p* < 0.01). In the analysis of T2 (vs. T0) (*I* ^2^ = 77%, *p* < 0.01), the “long‐term” follow‐up group (*I* ^2^ = 61%, *p* = 0.03) also showed lower heterogeneity compared to the “short‐term” follow‐up group (*I* ^2^ = 80%, *p* < 0.01). In contrast, in the analysis of hypertension (*I* ^2^ = 77%, *p* < 0.01), the “long‐term” follow‐up group exhibited higher heterogeneity (*I* ^2^ = 83%, *p* < 0.01) compared to the “short‐term” follow‐up group (*I* ^2^ = 68%, *p* < 0.01). Moreover, in the analysis of C1 (vs. C0) (*I* ^2^ = 89%, *p* < 0.01), no heterogeneity was found in the “moderate” group (*I* ^2^ = 0%, *p* = 0.38), whereas substantial heterogeneity was present in the “severe” group (*I* ^2^ = 77%, *p* < 0.01). These findings suggest that the heterogeneity may be attributed to differences in follow‐up periods, eligibility criteria and study endpoints.

This meta‐analysis has several limitations. First, the sources of heterogeneity were multifaceted, and the high heterogeneity persisted after both sensitivity and subgroup analysis for Alb, eGFR, Hb, MAP, SBP, SCr, 24‐h UPE, male (vs. female), C4, hypertension, age, UA, cholesterol, LY count, NLR, PLR, C1 (vs. C0), T1/T2 (vs. T0) and T2 (vs. T0). Second, considerable variation in follow‐up periods and study endpoints among the included studies may affect the overall findings. Third, kidney disease outcomes were not uniformly defined across studies, and the methodological quality of the included studies was variable. These inconsistencies, along with potential confounding factors, may have impacted the robustness and generalizability of the results.

## 5 Conclusions

In summary, this study confirms the predictive role of several established risk factors for disease progression, including higher MAP, higher DBP, higher SBP, hypertension, higher proteinuria, the increase in SCr, triglycerides, LDL‐C, C4, and male sex (vs. female). In contrast, increased levels of Alb, eGFR, Hb, and C3 were identified as protective factors in patients with IgAN. Owing to the highly variable nature of IgAN, predicting renal progression based solely on clinical data remains challenging. Furthermore, our findings indicate that C‐lesions, M‐lesions, S‐lesions, and T‐lesions (but not E‐lesions and C1) of the Oxford classification are strongly associated with IgAN progression.

## Author Contributions

**Dan Xu** and **Feifei Ge:** conception, design, administrative support. **Weiwei Liang**, **Lijiang Fang,** and **Dan Xu:** data analysis, interpretation. **Feifei Ge**, **Minjie Zhang**, and **Dan Xu:** manuscript writing, collection, assembly of data. All authors read and approved the final manuscript.

## Conflicts of Interest

The authors declare no conflicts of interest.

## Supporting information


**Table S1:** Quality scores of studies using Newcastle‐Ottawa Scale.

**Table S2:** Characteristics of the included studies.

## Data Availability

Requests for any data can be made to the authors.

## References

[1] K. N. Lai , S. C. W. Tang , F. P. Schena , et al., “IgA Nephropathy,” Nature Reviews Disease Primers 2 (2016): 16001, 10.1038/nrdp.2016.1. 27189177

[2] G. D'Amico , “Natural History of Idiopathic IgA Nephropathy and Factors Predictive of Disease Outcome,” Seminars in Nephrology 24, no. 3 (2004): 179–196, 10.1016/j.semnephrol.2004.01.001. 15156525

[3] I. S. D. Roberts , “Pathology of IgA Nephropathy: A Global Perspective,” Nephrology 29, no. S2 (2024): 71–74, 10.1111/nep.14343. 39327761

[4] D. C. Cattran , R. Coppo , H. T. Cook , et al., “The Oxford Classification of IgA Nephropathy: Rationale, Clinicopathological Correlations, and Classification,” Kidney International 76, no. 5 (2009): 534–545, 10.1038/ki.2009.243. 19571791

[5] Z. Abuduwupuer , Q. Lei , S. Liang , et al., “The Spectrum of Biopsy‐Proven Kidney Diseases, Causes, and Renal Outcomes in Acute Kidney Injury Patients,” Nephron 147, no. 9 (2023): 541–549, 10.1159/000530615. 37094563

[6] R. Coppo , “Clinical and Histological Risk Factors for Progression of IgA Nephropathy: An Update in Children, Young and Adult Patients,” Journal of Nephrology 30, no. 3 (2017): 339–346, 10.1007/s40620-016-0360-z. 27815919

[7] Y. Liu , W. Wei , C. Yu , et al., “Epidemiology and Risk Factors for Progression in Chinese Patients With IgA Nephropathy,” Medicina Clínica 157, no. 6 (2021): 267–273, 10.1016/j.medcli.2020.05.064. 32826075

[8] H. Trimarchi , J. Barratt , D. C. Cattran , et al., “Oxford Classification of IgA Nephropathy 2016: An Update From the IgA Nephropathy Classification Working Group,” Kidney International 91, no. 5 (2017): 1014–1021, 10.1016/j.kint.2017.02.003. 28341274

[9] M. F. S. Soares and I. S. D. Roberts , “Histologic Classification of IgA Nephropathy: Past, Present, and Future,” Seminars in Nephrology 38, no. 5 (2018): 477–484, 10.1016/j.semnephrol.2018.05.017. 30177019

[10] X. Zhu , H. Li , Y. Liu , et al., “Tubular Atrophy/Interstitial Fibrosis Scores of Oxford Classification Combinded With Proteinuria Level at Biopsy Provides Earlier Risk Prediction in IgA Nephropathy,” Scientific Reports 7, no. 1 (2017): 1100, 10.1038/s41598-017-01223-3. 28439112 PMC5430886

[11] M. Iwano and E. G. Neilson , “Mechanisms of Tubulointerstitial Fibrosis,” Current Opinion in Nephrology and Hypertension 13, no. 3 (2004): 279–284, 10.1097/00041552-200405000-00003. 15073485

[12] Y. Du , S. Chen , F. Wang , et al., “The Significance of Crescents on the Clinical Features and Outcomes of Primary Immunoglobin A Nephropathy,” Frontiers in Medicine 9 (2022): 864667, 10.3389/fmed.2022.864667. 35847826 PMC9276938

[13] J. Lv , S. Shi , D. Xu , et al., “Evaluation of the Oxford Classification of IgA Nephropathy: A Systematic Review and Meta‐Analysis,” American Journal of Kidney Diseases 62, no. 5 (2013): 891–899, 10.1053/j.ajkd.2013.04.021. 23820066

[14] S. J. Barbour , D. C. Cattran , S. J. Kim , et al., “Individuals of Pacific Asian Origin With IgA Nephropathy Have an Increased Risk of Progression to End‐Stage Renal Disease,” Kidney International 84, no. 5 (2013): 1017–1024, 10.1038/ki.2013.210. 23739233

[15] T. Bi , J. Zheng , J. Zhang , et al., “Serum Complement C4 Is an Important Prognostic Factor for IgA Nephropathy: A Retrospective Study,” BMC Nephrology 20, no. 1 (2019): 244, 10.1186/s12882-019-1420-0. 31272400 PMC6610919

[16] B. Descamps‐Latscha , V. Witko‐Sarsat , T. Nguyen‐Khoa , et al., “Early Prediction of IgA Nephropathy Progression: Proteinuria and AOPP Are Strong Prognostic Markers,” Kidney International 66, no. 4 (2004): 1606–1612, 10.1111/j.1523-1755.2004.00926.x. 15458457

[17] B. Faria , C. Henriques , A. C. Matos , M. R. Daha , M. Pestana , and M. Seelen , “Combined C4d and CD3 Immunostaining Predicts Immunoglobulin (Ig)A Nephropathy Progression,” Clinical and Experimental Immunology 179, no. 2 (2015): 354–361, 10.1111/cei.12461. 25267249 PMC4298411

[18] N. Farooqui , A. Subbiah , P. Chaturvedi , et al., “Association of Vitamin D Status With Disease Severity and Outcome in Indian Patients With IgA Nephropathy,” BMC Nephrology 24, no. 1 (2023): 15, 10.1186/s12882-023-03061-0. 36650464 PMC9843909

[19] K. Harada , Y. Akai , N. Kurumatani , M. Iwano , and Y. Saito , “Prognostic Value of Urinary Interleukin 6 in Patients With IgA Nephropathy: An 8‐Year Follow‐Up Study,” Nephron 92, no. 4 (2002): 824–826, 10.1159/000065465. 12399627

[20] S. J. Kim , H. M. Koo , B. J. Lim , et al., “Decreased Circulating C3 Levels and Mesangial C3 Deposition Predict Renal Outcome in Patients With IgA Nephropathy,” PLoS One 7, no. 7 (2012): e40495, 10.1371/journal.pone.0040495. 22792353 PMC3391269

[21] W. Le , S. Liang , Y. Hu , et al., “Long‐Term Renal Survival and Related Risk Factors in Patients With IgA Nephropathy: Results From a Cohort of 1155 Cases in a Chinese Adult Population,” Nephrology Dialysis Transplantation 27, no. 4 (2012): 1479–1485, 10.1093/ndt/gfr527. 21965586

[22] H. Li , W. Lu , X. Xie , et al., “Serum Phosphorus Might Be a Predictor of Kidney Disease Progression in IgA Nephropathy,” Kidney & Blood Pressure Research 49, no. 1 (2024): 20–26, 10.1159/000535608. 38048756

[23] Q. Li , P. Chen , S. Shi , et al., “Neutrophil‐to‐Lymphocyte Ratio as an Independent Inflammatory Indicator of Poor Prognosis in IgA Nephropathy,” International Immunopharmacology 87 (2020): 106811, 10.1016/j.intimp.2020.106811. 32711375

[24] Y. Li , S. Jiang , H. Gao , Y. Yang , X. Liu , and W. Li , “Factors Associated With the Progression of Mesangial Lesions in IgA Nephropathy: A Comparative Analysis of Renal Re‐Biopsies,” Frontiers in Endocrinology 13 (2022): 1004289, 10.3389/fendo.2022.1004289. 36479219 PMC9719920

[25] D. Liu , J. You , Y. Liu , et al., “Serum Immunoglobulin G Provides Early Risk Prediction in Immunoglobulin A Nephropathy,” International Immunopharmacology 66 (2019): 13–18, 10.1016/j.intimp.2018.10.044. 30415190

[26] J. Liu , S. Duan , P. Chen , et al., “Development and Validation of a Prognostic Nomogram for IgA Nephropathy,” Oncotarget 8, no. 55 (2017): 94371–94381, 10.18632/oncotarget.21721. 29212234 PMC5706880

[27] L. Liu , L. Zhu , J. Zheng , et al., “Development and Assessment of a Predictive Nomogram for the Progression of IgA Nephropathy,” Scientific Reports 8, no. 1 (2018): 7309, 10.1038/s41598-018-25653-9. 29743598 PMC5943256

[28] Y. Liu , W. Wei , C. Yu , et al., “Epidemiology and Risk Factors for Progression in Chinese Patients With IgA Nephropathy,” Medicina Clínica 157, no. 6 (2021): 267–273, 10.1016/j.medcli.2020.05.064. 32826075

[29] F. Ma , L. Liu , R. Dong , et al., “Renal Survival and Risk Factors in IgA Nephropathy With Crescents,” International Urology and Nephrology 52, no. 8 (2020): 1507–1516, 10.1007/s11255-020-02457-3. 32533530

[30] R. Mohd , N. E. Mohammad Kazmin , R. Abdul Cader , et al., “Long Term Outcome of Immunoglobulin A (IgA) Nephropathy: A Single Center Experience,” PLoS One 16, no. 4 (2021): e0249592, 10.1371/journal.pone.0249592. 33831052 PMC8031432

[31] T. Moriyama , K. Nakayama , C. Iwasaki , et al., “Severity of Nephrotic IgA Nephropathy According to the Oxford Classification,” International Urology and Nephrology 44, no. 4 (2012): 1177–1184, 10.1007/s11255-011-0109-5. 22231129

[32] T. Moriyama , M. Itabashi , T. Takei , et al., “High Uric Acid Level Is a Risk Factor for Progression of IgA Nephropathy With Chronic Kidney Disease Stage G3a,” Journal of Nephrology 28, no. 4 (2015): 451–456, 10.1007/s40620-014-0154-0. 25355499

[33] Y. Ouyang , J. Xie , M. Yang , et al., “Underweight Is an Independent Risk Factor for Renal Function Deterioration in Patients With IgA Nephropathy,” PLoS One 11, no. 9 (2016): e0162044, 10.1371/journal.pone.0162044. 27611091 PMC5017745

[34] M. Pan , Q. Zhou , S. Zheng , et al., “Serum C3/C4 Ratio Is a Novel Predictor of Renal Prognosis in Patients With IgA Nephropathy: A Retrospective Study,” Immunologic Research 66, no. 3 (2018): 381–391, 10.1007/s12026-018-8995-6. 29850970

[35] N. Pană , G. Ștefan , T. Popa , O. Ciurea , S. H. Stancu , and C. Căpușă , “Prognostic Value of Inflammation Scores and Hematological Indices in IgA and Membranous Nephropathies: An Exploratory Study,” Medicina (Kaunas) 60, no. 8 (2024): 1191, 10.3390/medicina60081191. 39202473 PMC11356348

[36] G. Y. Park , C. H. Yu , J. S. Kim , et al., “Plasma Neutrophil Gelatinase‐Associated Lipocalin as a Potential Predictor of Adverse Renal Outcomes in Immunoglobulin A Nephropathy,” Korean Journal of Internal Medicine 30, no. 3 (2015): 345–353, 10.3904/kjim.2015.30.3.345. 25995665 PMC4438289

[37] H. P. E. Peters , F. Waanders , E. Meijer , et al., “High Urinary Excretion of Kidney Injury Molecule‐1 Is an Independent Predictor of End‐Stage Renal Disease in Patients With IgA Nephropathy,” Nephrology Dialysis Transplantation 26, no. 11 (2011): 3581–3588, 10.1093/ndt/gfr135. 21467131

[38] C. Qi , X. Liu , J. Mao , et al., “The Time‐Averaged Serum Uric Acid Can Better Predict the Prognosis of IgA Nephropathy,” Nutrition, Metabolism, and Cardiovascular Diseases 35, no. 3 (2025): 103800, 10.1016/j.numecd.2024.103800. 39674719

[39] H. Rhee , N. Shin , M. J. Shin , et al., “High Serum and Urine Neutrophil Gelatinase‐Associated Lipocalin Levels Are Independent Predictors of Renal Progression in Patients With Immunoglobulin A Nephropathy,” Korean Journal of Internal Medicine 30, no. 3 (2015): 354–361, 10.3904/kjim.2015.30.3.354. 25995666 PMC4438290

[40] N. Saleem , H. Nasir , F. Anwar , et al., “To Evaluate the Utility of Oxford Classification in Predicting Renal Outcome in IgA Nephropathy Patients,” International Urology and Nephrology 56, no. 1 (2024): 345–353, 10.1007/s11255-023-03685-z. 37378850

[41] D. H. Shin , B. J. Lim , I. M. Han , et al., “Glomerular IgG Deposition Predicts Renal Outcome in Patients With IgA Nephropathy,” Modern Pathology 29, no. 7 (2016): 743–752, 10.1038/modpathol.2016.77. 27102346

[42] J. Tan , G. Song , S. Wang , et al., “Platelet‐to‐Albumin Ratio: A Novel IgA Nephropathy Prognosis Predictor,” Frontiers in Immunology 13 (2022): 842362, 10.3389/fimmu.2022.842362. 35664006 PMC9162245

[43] L. Tan , Y. Tang , G. Pei , et al., “A Multicenter, Prospective, Observational Study to Determine Association of Mesangial C1q Deposition With Renal Outcomes in IgA Nephropathy,” Scientific Reports 11, no. 1 (2021): 5467, 10.1038/s41598-021-84715-7. 33750830 PMC7943768

[44] T. Tang , L. Li , Y. Chen , et al., “High Serum Cystatin C Is an Independent Risk Factor for Poor Renal Prognosis in IgA Nephropathy,” Nan fang yi ke da xue xue bao = Journal of Southern Medical University 45, no. 2 (2025): 379–386, 10.12122/j.issn.1673-4254.2025.02.19. 40031982 PMC11875852

[45] Z. Y. Tian , A. M. Li , L. Chu , J. Hu , X. Xie , and H. Zhang , “Prognostic Value of Low‐Density Lipoprotein Cholesterol in IgA Nephropathy and Establishment of Nomogram Model,” Frontiers in Endocrinology 14 (2023): 1037773, 10.3389/fendo.2023.1037773. 36843611 PMC9950098

[46] D. D. Torres , M. Rossini , C. Manno , et al., “The Ratio of Epidermal Growth Factor to Monocyte Chemotactic Peptide‐1 in the Urine Predicts Renal Prognosis in IgA Nephropathy,” Kidney International 73, no. 3 (2008): 327–333, 10.1038/sj.ki.5002621. 17943082

[47] M. Walsh , A. Sar , D. Lee , et al., “Histopathologic Features Aid in Predicting Risk for Progression of IgA Nephropathy,” Clinical Journal of the American Society of Nephrology 5, no. 3 (2010): 425–430, 10.2215/cjn.06530909. 20089495 PMC2827572

[48] S. Wang , L. Dong , G. Pei , et al., “High Neutrophil‐to‐Lymphocyte Ratio Is an Independent Risk Factor for End Stage Renal Diseases in IgA Nephropathy,” Frontiers in Immunology 12 (2021): 700224, 10.3389/fimmu.2021.700224. 34456912 PMC8387559

[49] Y. Wang , S. Jiang , D. Di , et al., “The Prognostic Role of Activation of the Complement Pathways in the Progression of Advanced IgA Nephropathy to End‐Stage Renal Disease,” BMC Nephrology 25, no. 1 (2024): 387, 10.1186/s12882-024-03832-3. 39478440 PMC11523594

[50] W. Ying , S. Shang , S. Jiang , G. Zou , H. Gao , and W. Li , “Complement C3a/C3aR and C5a/C5aR Deposits Accelerate the Progression of Advanced IgA Nephropathy to End‐Stage Renal Disease,” Clinical and Experimental Medicine 24, no. 1 (2024): 139, 10.1007/s10238-024-01410-3. 38951265 PMC11217045

[51] S. Worawichawong , S. Plumworasawat , W. Liwlompaisan , et al., “Distribution Pattern of Mesangial C4d Deposits as Predictor of Kidney Failure in IgA Nephropathy,” PLoS One 16, no. 6 (2021): e0252638, 10.1371/journal.pone.0252638. 34081719 PMC8174712

[52] D. Wu , X. Li , X. Yao , et al., “Mesangial C3 Deposition and Serum C3 Levels Predict Renal Outcome in IgA Nephropathy,” Clinical and Experimental Nephrology 25, no. 6 (2021): 641–651, 10.1007/s10157-021-02034-7. 33620604

[53] M. Xia , D. Liu , L. Peng , et al., “Coagulation Parameters Are Associated With the Prognosis of Immunoglobulin a Nephropathy: A Retrospective Study,” BMC Nephrology 21, no. 1 (2020): 447, 10.1186/s12882-020-02111-1. 33109129 PMC7590710

[54] J. Xie , J. Lv , W. Wang , et al., “Kidney Failure Risk Prediction Equations in IgA Nephropathy: A Multicenter Risk Assessment Study in Chinese Patients,” American Journal of Kidney Diseases 72, no. 3 (2018): 371–380, 10.1053/j.ajkd.2018.01.043. 29555434

[55] J. Xie , K. Kiryluk , W. Wang , et al., “Predicting Progression of IgA Nephropathy: New Clinical Progression Risk Score,” PLoS One 7, no. 6 (2012): e38904, 10.1371/journal.pone.0038904. 22719981 PMC3375310

[56] Y. Xing , H. Yu , H. Li , et al., “Glomerular IgG Deposition Predicts Kidney Disease Progression in IgA Nephropathy,” Heliyon 10, no. 7 (2024): e28509, 10.1016/j.heliyon.2024.e28509. 38571652 PMC10988001

[57] X. Xu , X. Huang , Y. Chen , et al., “The Role of Urine IgG in the Progression of IgA Nephropathy With a High Proportion of Global Glomerulosclerosis,” International Urology and Nephrology 54, no. 2 (2022): 323–330, 10.1007/s11255-021-02858-y. 33871780

[58] W. Yang , R. Zhu , J. Zheng , et al., “Glomerular Deposition of Fibrinogen Predicts Good Prognosis of IgA Nephropathy: A Single‐Center Cohort Study,” International Urology and Nephrology 55, no. 7 (2023): 1857–1864, 10.1007/s11255-023-03501-8. 36787086 PMC10293415

[59] Y. Yang , X. Tang , Y. Yang , et al., “Glomerular C4 Deposition and Glomerulosclerosis Predict Worse Renal Outcomes in Chinese Patients With IgA Nephropathy,” Renal Failure 42, no. 1 (2020): 629–637, 10.1080/0886022x.2020.1786400. 32660366 PMC7470092

[60] S. Y. Yoon , J. S. Kim , S. W. Jung , et al., “Clinical Significance of Urinary Inflammatory Biomarkers in Patients With IgA Nephropathy,” BMC Nephrology 25, no. 1 (2024): 142, 10.1186/s12882-024-03574-2. 38649936 PMC11036669

[61] G. Yu , J. Cheng , Y. Jiang , H. Li , X. Li , and J. Chen , “Serum Phosphorus and Calcium Levels, and Kidney Disease Progression in Immunoglobulin A Nephropathy,” Clinical Kidney Journal 14, no. 9 (2021): 2108–2113, 10.1093/ckj/sfab002. 34476094 PMC8406074

[62] Z. Yu , Y. Qin , J. Yuan , J. Zhao , and S. Sun , “Retrospective Analysis of the Effect of Uric Acid on the Prognosis of Immunoglobulin A Nephropathy With Stage 3‐4 Chronic Kidney Disease],” Sichuan da xue xue bao. Yi xue ban = Journal of Sichuan University Medical Science Edition 54, no. 6 (2023): 1121–1127, 10.12182/20231160505. 38162075 PMC10752786

[63] N. Zagorec , I. Horvatić , D. Kasumović , et al., “C4d Is an Independent Predictor of the Kidney Failure in Primary IgA Nephropathy,” Journal of Clinical Medicine 13, no. 17 (2024): 5338, 10.3390/jcm13175338. 39274551 PMC11395978

[64] Y. Zhai , S. Sun , W. Zhang , and H. Tian , “The Prognostic Value of the Systemic Immune Inflammation Index in Patients With IgA Nephropathy,” Renal Failure 46, no. 2 (2024): 2381613, 10.1080/0886022x.2024.2381613. 39039867 PMC11268256

[65] J. Zhang , C. Chen , Q. Zhou , et al., “Elevated Serum Fibrinogen Level Is an Independent Risk Factor for IgA Nephropathy,” Oncotarget 8, no. 58 (2017): 99125–99135, 10.18632/oncotarget.21702. 29228758 PMC5716798

[66] Y. Zhao , L. Zhu , L. Liu , S. Shi , J. Lv , and H. Zhang , “Measures of Urinary Protein and Albumin in the Prediction of Progression of IgA Nephropathy,” Clinical Journal of the American Society of Nephrology 11, no. 6 (2016): 947–955, 10.2215/cjn.10150915. 27026518 PMC4891752

[67] Y. Nihei and D. Kitamura , “Pathogenesis of IgA Nephropathy as a Tissue‐Specific Autoimmune Disease,” International Immunology 37, no. 2 (2024): 75–81, 10.1093/intimm/dxae047. 39066568

[68] D. Petrou , P. Kalogeropoulos , G. Liapis , and S. Lionaki , “IgA Nephropathy: Current Treatment and New Insights,” Antibodies 12, no. 2 (2023): 40, 10.3390/antib12020040. 37366657 PMC10294861

[69] Y. Rui , Z. Yang , Z. Zhai , C. Zhao , and L. Tang , “The Predictive Value of Oxford MEST‐C Classification to Immunosuppressive Therapy of IgA Nephropathy,” International Urology and Nephrology 54, no. 4 (2022): 959–967, 10.1007/s11255-021-02974-9. 34383207

[70] E. Stamellou , C. Seikrit , S. C. W. Tang , et al., “IgA Nephropathy,” Nature Reviews Disease Primers 9, no. 1 (2023): 67, 10.1038/s41572-023-00476-9. 38036542

[71] Q. Binet , S. Aydin , J. P. Lengele , and J. F. Cambier , “Lessons for the Clinical Nephrologist: An Uncommon Cause of Pulmonary‐Renal Syndrome,” Journal of Nephrology 34, no. 3 (2021): 935–938, 10.1007/s40620-020-00846-6. 32870493

[72] L. J. Appel , J. T. Wright, Jr. , T. Greene , et al., “Intensive Blood‐Pressure Control in Hypertensive Chronic Kidney Disease,” New England Journal of Medicine 363, no. 10 (2010): 918–929, 10.1056/NEJMoa0910975. 20818902 PMC3662974

[73] C. Li , K. Chen , V. Cornelius , et al., “Applicability and Cost‐Effectiveness of the Systolic Blood Pressure Intervention Trial (SPRINT) in the Chinese Population: A Cost‐Effectiveness Modeling Study,” PLoS Medicine 18, no. 3 (2021): e1003515, 10.1371/journal.pmed.1003515. 33661907 PMC7971845

[74] M. Q. Mo , L. Pan , Q. M. Lu , Q. L. Li , and Y. H. Liao , “The Association of the CMIP rs16955379 Polymorphism With Dyslipidemia and the Clinicopathological Features of IgA Nephropathy,” International Journal of Clinical and Experimental Pathology 11, no. 10 (2018): 5008–5023. 31949578 PMC6962923

[75] J. Syrjänen , J. Mustonen , and A. Pasternack , “Hypertriglyceridaemia and Hyperuricaemia Are Risk Factors for Progression of IgA Nephropathy,” Nephrology Dialysis Transplantation 15, no. 1 (2000): 34–42, 10.1093/ndt/15.1.34. 10607765

[76] S. Agrawal , J. J. Zaritsky , A. Fornoni , and W. E. Smoyer , “Dyslipidaemia in Nephrotic Syndrome: Mechanisms and Treatment,” Nature Reviews Nephrology 14, no. 1 (2018): 57–70, 10.1038/nrneph.2017.155. 29176657 PMC5770189

[77] L. Gyebi , Z. Soltani , and E. Reisin , “Lipid Nephrotoxicity: New Concept for an Old Disease,” Current Hypertension Reports 14, no. 2 (2012): 177–181, 10.1007/s11906-012-0250-2. 22290079

[78] F. Irie , H. Iso , T. Sairenchi , et al., “The Relationships of Proteinuria, Serum Creatinine, Glomerular Filtration Rate With Cardiovascular Disease Mortality in Japanese General Population,” Kidney International 69, no. 7 (2006): 1264–1271, 10.1038/sj.ki.5000284. 16501489

[79] M. Canney , S. J. Barbour , Y. Zheng , et al., “Quantifying Duration of Proteinuria Remission and Association With Clinical Outcome in IgA Nephropathy,” Journal of the American Society of Nephrology 32, no. 2 (2021): 436–447, 10.1681/asn.2020030349. 33514642 PMC8054888

[80] E. Tringali , D. Vetrano , F. Tondolo , et al., “Role of Serum Complement C3 and C4 on Kidney Outcomes in IgA Nephropathy,” Scientific Reports 14, no. 1 (2024): 16224, 10.1038/s41598-024-65857-w. 39003309 PMC11246413

[81] M. Pan , J. Zhang , Z. Li , et al., “Increased C4 and Decreased C3 Levels Are Associated With a Poor Prognosis in Patients With Immunoglobulin A Nephropathy: A Retrospective Study,” BMC Nephrology 18, no. 1 (2017): 231, 10.1186/s12882-017-0658-7. 28697742 PMC5505039

[82] B. Zhu , C. F. Zhu , Y. Lin , et al., “Clinical Characteristics of IgA Nephropathy Associated With Low Complement 4 Levels,” Renal Failure 37, no. 3 (2015): 424–432, 10.3109/0886022x.2014.994408. 25539484

[83] Y. H. Geng , Z. Zhang , J. J. Zhang , et al., “Hyperuricemia Aggravates the Progression of IgA Nephropathy,” International Urology and Nephrology 54, no. 9 (2022): 2227–2237, 10.1007/s11255-022-03125-4. 35072913

[84] L. Lu , B. Li , G. Dai , Y. Feng , and H. Feng , “Association Between Hyperuricaemia and Clinical Pathological Characteristics of Patients With IgA Nephropathy,” Journal of the Pakistan Medical Association 71, no. 8 (2021): 1930–1934, 10.47391/jpma.413. 34418003

[85] G. Yu , L. Guo , J. Dong , et al., “Persistent Hematuria and Kidney Disease Progression in IgA Nephropathy: A Cohort Study,” American Journal of Kidney Diseases 76, no. 1 (2020): 90–99, 10.1053/j.ajkd.2019.11.008. 32197881

[86] Q. Wang , J. Zhang , W. Dou , H. Zeng , P. Shi , and J. Wu , “Impact of Body Mass Index on Primary Immunoglobulin A Nephropathy Prognosis: A Systematic Review and Meta‐Analysis,” International Urology and Nephrology 54, no. 5 (2022): 1067–1078, 10.1007/s11255-021-02978-5. 34383206

[87] K. Iseki , Y. Ikemiya , K. Kinjo , T. Inoue , C. Iseki , and S. Takishita , “Body Mass Index and the Risk of Development of End‐Stage Renal Disease in a Screened Cohort,” Kidney International 65, no. 5 (2004): 1870–1876, 10.1111/j.1523-1755.2004.00582.x. 15086929

[88] T. R. Oh , S. H. Song , H. S. Choi , et al., “The Association Between Serum Hemoglobin and Renal Prognosis of IgA Nephropathy,” Journal of Clinical Medicine 10, no. 2 (2021): 363, 10.3390/jcm10020363. 33478025 PMC7835832

[89] M. Nangaku , “Chronic Hypoxia and Tubulointerstitial Injury: A Final Common Pathway to End‐Stage Renal Failure,” Journal of the American Society of Nephrology 17, no. 1 (2006): 17–25, 10.1681/asn.2005070757. 16291837

[90] W. G. Murphy , “The Sex Difference in Haemoglobin Levels in Adults—Mechanisms, Causes, and Consequences,” Blood Reviews 28, no. 2 (2014): 41–47, 10.1016/j.blre.2013.12.003. 24491804

[91] S. J. Barbour , G. Espino‐Hernandez , H. N. Reich , et al., “The MEST Score Provides Earlier Risk Prediction in lgA Nephropathy,” Kidney International 89, no. 1 (2016): 167–175, 10.1038/ki.2015.322. 26759049

[92] S. Park , H. Go , C. H. Baek , et al., “Clinical Importance of the Updated Oxford Classification in Allograft IgA Nephropathy,” American Journal of Transplantation 19, no. 10 (2019): 2855–2864, 10.1111/ajt.15400. 31017369

[93] A. Chakera , C. MacEwen , S. S. Bellur , L. Chompuk , D. Lunn , and I. S. D. Roberts , “Prognostic Value of Endocapillary Hypercellularity in IgA Nephropathy Patients With No Immunosuppression,” Journal of Nephrology 29, no. 3 (2016): 367–375, 10.1007/s40620-015-0227-8. 26318019

[94] B. J. Lim , D. J. Joo , M. S. Kim , et al., “Usefulness of Oxford Classification in Assessing Immunoglobulin A Nephropathy After Transplantation,” Transplantation 95, no. 12 (2013): 1491–1497, 10.1097/TP.0b013e318291de65. 23677050

[95] R. Coppo , S. Troyanov , S. Bellur , et al., “Validation of the Oxford Classification of IgA Nephropathy in Cohorts With Different Presentations and Treatments,” Kidney International 86, no. 4 (2014): 828–836, 10.1038/ki.2014.63. 24694989 PMC4184028

[96] S. F. Shi , S. X. Wang , L. Jiang , et al., “Pathologic Predictors of Renal Outcome and Therapeutic Efficacy in IgA Nephropathy: Validation of the Oxford Classification,” Clinical Journal of the American Society of Nephrology 6, no. 9 (2011): 2175–2184, 10.2215/cjn.11521210. 21852672 PMC3358999

[97] R. Xu , Z. Li , T. Cao , et al., “The Association of the Oxford Classification Score With Longitudinal Estimated Glomerular Filtration Rate Decline in Patients With Immunoglobulin A Nephropathy: A Mixed‐Method Study,” International Journal of General Medicine 14 (2021): 2655–2663, 10.2147/ijgm.S313333. 34177274 PMC8219302

