# Supplementary material for: Risk Factors of Disease Progression in IgA Nephropathy: A Systematic Review and Meta‐Analysis
Source: Immun Inflamm Dis. 2026 Feb 27;14(2):e70393. doi: 10.1002/iid3.70393 (PMC12946928; doi:10.1002/iid3.70393)
Supplement: Supplementary file 1 — Table S1: Quality scores of studies using Newcastle‐Ottawa Scale. [file IID3-14-e70393-s002.doc]

Table S1. Quality scores of studies using Newcastle-Ottawa Scale

| Study | Selection | | | | Comparability | Outcome | | | NOS |
| --- | --- | --- | --- | --- | --- | --- | --- | --- | --- |
|  | Q1 | Q2 | Q3 | Q4 | Q5 | Q6 | Q7 | Q8 | Overall score |
| Barbour SJ^14^ 2013 | 1 | 1 | 1 | 1 | 1 | 1 | 1 | 1 | 8 |
| Bi TD^15^ 2019 | 1 | 1 | 1 | 1 | 1 | 1 | 1 | 1 | 8 |
| Descamps-Latscha B^16^ 2004 | 1 | 1 | 1 | 1 | 1 | 1 | 1 | 0 | 7 |
| Faria B^17^ 2015 | 1 | 1 | 1 | 1 | 1 | 1 | 1 | 1 | 8 |
| Farooqui N^18^ 2023 | 1 | 1 | 1 | 1 | 1 | 1 | 1 | 0 | 7 |
| Harada K^19^ 2002 | 1 | 1 | 1 | 1 | 1 | 1 | 1 | 0 | 7 |
| Kim SJ^20^ 2012 | 1 | 1 | 1 | 1 | 1 | 1 | 1 | 1 | 8 |
| Le W^21^ 2012 | 1 | 1 | 1 | 1 | 1 | 1 | 1 | 1 | 8 |
| Li H^22^ 2024 | 1 | 1 | 1 | 1 | 1 | 1 | 1 | 1 | 8 |
| Li Q^23^ 2020 | 1 | 1 | 1 | 1 | 1 | 1 | 0 | 0 | 6 |
| Li Y^24^ 2022 | 1 | 1 | 1 | 1 | 1 | 1 | 0 | 0 | 6 |
| Liu D^25^ 2019 | 1 | 1 | 1 | 0 | 1 | 1 | 0 | 1 | 6 |
| Liu J^26^ 2017 | 1 | 1 | 1 | 1 | 0 | 1 | 0 | 0 | 5 |
| Liu LL^27^ 2018 | 1 | 1 | 1 | 1 | 0 | 1 | 0 | 0 | 5 |
| Liu Y^28^ 2021 | 1 | 1 | 1 | 1 | 0 | 1 | 1 | 0 | 6 |
| Ma F^29^ 2020 | 1 | 1 | 1 | 1 | 1 | 1 | 0 | 0 | 6 |
| Mohd R^30^ 2021 | 1 | 1 | 1 | 1 | 1 | 1 | 1 | 0 | 7 |
| Moriyama T^31^ 2012 | 1 | 1 | 1 | 1 | 1 | 1 | 0 | 0 | 6 |
| Moriyama T^32^ 2015 | 1 | 1 | 1 | 1 | 1 | 1 | 0 | 0 | 6 |
| Ouyang Y^33^ 2016 | 1 | 1 | 1 | 1 | 1 | 1 | 1 | 1 | 8 |
| Pan M^34^ 2018 | 1 | 1 | 1 | 1 | 1 | 1 | 1 | 0 | 7 |
| Pană N^35^ 2024 | 1 | 1 | 1 | 1 | 1 | 1 | 1 | 0 | 7 |
| Park GY^36^ 2015 | 1 | 1 | 1 | 1 | 1 | 1 | 0 | 0 | 6 |
| Peters HP^37^ 2011 | 1 | 0 | 1 | 1 | 0 | 1 | 0 | 0 | 4 |
| Qi C^38^ 2025 | 1 | 1 | 1 | 1 | 1 | 1 | 1 | 0 | 7 |
| Rhee H^39^ 2015 | 1 | 1 | 1 | 1 | 1 | 1 | 0 | 0 | 6 |
| Saleem N^40^ 2024 | 1 | 1 | 1 | 1 | 1 | 1 | 1 | 0 | 7 |
| Shin DH^41^ 2016 | 1 | 1 | 1 | 1 | 1 | 1 | 1 | 1 | 8 |
| Tan J^42^ 2022 | 1 | 1 | 1 | 1 | 1 | 1 | 0 | 1 | 7 |
| Tan L^43^ 2021 | 1 | 1 | 1 | 1 | 1 | 1 | 1 | 1 | 8 |
| Tang T^44^ 2025 | 1 | 1 | 1 | 1 | 1 | 1 | 1 | 0 | 7 |
| Tian ZY^45^ 2023 | 1 | 1 | 1 | 1 | 1 | 1 | 1 | 1 | 8 |
| Torres DD^46^ 2008 | 1 | 1 | 1 | 1 | 1 | 1 | 0 | 0 | 6 |
| Walsh M^47^ 2010 | 1 | 1 | 1 | 1 | 1 | 1 | 1 | 1 | 8 |
| Wang S^48^ 2021 | 1 | 1 | 1 | 1 | 1 | 1 | 1 | 1 | 8 |
| Wang Y^49^ 2024 | 1 | 1 | 1 | 1 | 1 | 1 | 1 | 0 | 7 |
| Wang Ying^50^ 2024 | 1 | 1 | 1 | 1 | 1 | 1 | 1 | 1 | 8 |
| Worawichawong S^51^ 2021 | 1 | 1 | 1 | 1 | 1 | 1 | 0 | 0 | 6 |

| Study | Selection | | | | Comparability | Outcome | | | NOS |
| --- | --- | --- | --- | --- | --- | --- | --- | --- | --- |
|  | Q1 | Q2 | Q3 | Q4 | Q5 | Q6 | Q7 | Q8 | Overall score |
| Wu D^52^ 2021 | 1 | 1 | 1 | 1 | 1 | 1 | 0 | 0 | 6 |
| Xia M^53^ 2020 | 1 | 1 | 1 | 1 | 1 | 1 | 1 | 0 | 7 |
| Xie J^54^ 2018 | 1 | 1 | 1 | 1 | 1 | 1 | 1 | 0 | 7 |
| Xie J^55^ 2012 | 1 | 1 | 1 | 1 | 1 | 1 | 1 | 0 | 7 |
| Xing Y^56^ 2024 | 1 | 1 | 1 | 1 | 1 | 1 | 1 | 1 | 8 |
| Xu X^57^ 2022 | 1 | 1 | 1 | 1 | 1 | 1 | 1 | 0 | 7 |
| Yang WG^58^ 2023 | 1 | 1 | 1 | 1 | 1 | 1 | 1 | 1 | 8 |
| Yang Y^59^ 2020 | 1 | 1 | 1 | 1 | 1 | 1 | 1 | 1 | 8 |
| Yoon SY^60^ 2024 | 1 | 0 | 1 | 1 | 1 | 1 | 1 | 0 | 6 |
| Yu G^61^ 2021 | 1 | 0 | 1 | 1 | 1 | 1 | 1 | 0 | 6 |
| Yu Z^62^ 2023 | 1 | 1 | 1 | 1 | 1 | 1 | 0 | 0 | 6 |
| Zagorec N^63^ 2024 | 1 | 1 | 1 | 1 | 1 | 1 | 1 | 0 | 7 |
| Zhai Y^64^ 2024 | 1 | 1 | 1 | 1 | 1 | 1 | 1 | 0 | 7 |
| Zhang J^65^ 2017 | 1 | 1 | 1 | 1 | 1 | 1 | 1 | 0 | 7 |
| Zhao YF^66^ 2016 | 1 | 1 | 1 | 1 | 1 | 1 | 1 | 1 | 8 |

Notes: Q1. Representativeness of the exposed cohort; Q2. Selection of the non exposed cohort; Q3. Ascertainment of exposure; Q4. Demonstration that outcomes was not present at start of study; Q5. Comparability on the basis of the design or analysis; Q6. Assessment of outcome; Q7. Adequate follow-up duration; Q8. Adequate follow-up rate
