# Supplementary material for: Risk Factors of Disease Progression in IgA Nephropathy: A Systematic Review and Meta‐Analysis
Source: Immun Inflamm Dis. 2026 Feb 27;14(2):e70393. doi: 10.1002/iid3.70393 (PMC12946928; doi:10.1002/iid3.70393)
Supplement: Supplementary file 2 — Table S2: Characteristics of the included studies. [file IID3-14-e70393-s001.doc]

| **Author/ Year** | **Study design**  **(Quality score)** | **Primary outcome** | **Description of the outcome events** | **General demographic characteristics** | | | |
| --- | --- | --- | --- | --- | --- | --- | --- |
|  |  |  |  | ***n*** | **Average age (years)^a^** | **F/M ratio** | **Follow-up periods** |
| Barbour SJ^14^ 2013^a^ | Retrospective cohort study | ESRD | 1. Dialysis or transplantation | 669 | Mean±SD 39.7±13.2 | 251/418 | Median (IQR)  46.4 months (17.9, 95.5) |
|  |  |  | 2. eGFR< 15 ml/min/1.73m^2^ |  |  |  |  |
|  |  |  | 3. 50% Reduction in eGFR |  |  |  |  |
|  |  |  | 4. Death |  |  |  |  |
| Bi TD^15^ 2019^a^ | Retrospective cohort study | Composite event | 1. ≥50% reduction in eGFR | 1052 | Mean±SD 37±13 | 527/525 | More than 1 year and  complete information |
|  |  |  | 2. ≥50% reduction in ESRD (eGFR was less than 15 ml/min/1.73m^2^ or renal replacement therapy was initiated) |  |  |  |  |
|  |  |  | 3. Death |  |  |  |  |
| Descamps-Latscha B^16^ 2004 | Prospective cohort study | Renal end point (as halving of baseline Ccr) | 1. A decrease in Ccr less than 50% at end of follow-up  were classified as group I | 120 | Mean±SD 39.9±15.5 | 29/91 | Mean±SD 5.4±2.5 years (range 1.2-7.9) |
|  |  |  | 2. Decline in Ccr was 50% or more were classified as group II. |  |  |  |  |
|  |  |  | 3. The rate of progression of renal failure was expressed as the yearly rate of decline in Ccr between entry in the study and end of follow-up or start of dialysis. |  |  |  |  |
| Faria B^17^ 2015 | Retrospective cohort study | Progressive kidney disease | 1. A decline of at least 50% in the eGFR | 74 | Median (IQR) 39 (18,78) | 24/50 | Median  4 years (range 0.3–10) |
|  |  |  | 2. ESRD (defined as chronic dialysis or renal transplantation or if the patient died) |  |  |  |  |
| Farooqui N^18^ 2023 | Retrospective cohort study | Renal disease progression | 1. At least 50% decline in eGFR | 105 | Mean±SD 34±10.6 | 32/73 | Median 21.5 Months (range 6-56) |
|  |  |  | 2. Progression to ESKD (eGFR < 10 ml/min/1.73m^2^ or requiring renal replacement therapy) |  |  |  |  |
| Harada K^19^ 2002 | Retrospective cohort study | Deterioration of renal function | 1. Ccr of less than 60 ml/min (range: 20-56 ml/min) (progressor group). | 59 | Mean±SD 34.6±11.3 | 33/26 | Mean±SD 8.07±1.72 years |
|  |  |  | 2. Ccr remained above 80 ml/min and percent reduction of Ccr is less than 10% (non-progressor group) |  |  |  |  |
| Kim SJ^20^ 2012 | Observational cohort study | ESRD and a doubling of the baseline serum creatinine (D-SCr) | 1. ESRD (initiation of renal replacement therapy including permanent hemodialysis, peritoneal dialysis, or renal transplantation) | 343 | Mean±SD 34.5±11.7 | 184/159 | Mean±SD 53.7±30.1 months |
|  |  |  | 2. A doubling of the baseline serum creatinine (D-SCr) |  |  |  |  |
| Le W^21^ 2012 | Retrospective cohort study | Combined event | 1. ESRD (eGFR <15 mL/min/1.73m^2^, initiation of dialysis or transplantation) | 1155 | Mean±SD 34±9 | 581/574 | Median (IQR) 5.4 years (4.1, 7.2) |
|  |  |  | 2. 50% reduction in renal function (the rate of renal function decline was expressed as the slope of eGFR) |  |  |  |  |
| Li H^22^ 2024 | Retrospective cohort study | Composite endpoint | 1. A 30% decrease in the eGFR | 247 | Mean±SD 36.0±12.4 | 112/135 | Mean±SD 35.8±18.8 months |
|  |  |  | 2. ESKD was defined as an eGFR <15 mL/min/1.73 m^2^ or the need for KRT (including hemodialysis, peritoneal dialysis, or kidney transplantation) |  |  |  |  |
| Li Q^23^ 2020 | Retrospective cohort study | Composite endpoint | 1. A 30% eGFR decline | 1151 | Median (IQR)  34.0 (27.0, 42.0) | 577/574 | Median (IQR) 45.0 months (24.0, 80.0) |
|  |  |  | 2. ESKD (eGFR < 15 ml/min per 1.73 m^2^ or the need for renal replacement therapy, including hemodialysis, peritoneal dialysis, or kidney transplantation) |  |  |  |  |

**Table S2 Characteristics of the included studies**

**Table S2 (Continued)**

| **Author/ Year** | **Study design**  **(Quality score)** | **Primary outcome** | **Description of the outcome events** | **General demographic characteristics** | | | |
| --- | --- | --- | --- | --- | --- | --- | --- |
|  |  |  |  | ***n*** | **Average age (years)^a^** | **F/M ratio** | **Follow-up periods** |
| Li Y^24^ 2022 | Retrospective cohort study | The progression of mesangial lesions | 1. Progressive (ratio > 1.1; hyperplasia > 10%) (Progression of mesangial hyperplasia was defined as a ratio of mesangial cell proliferation scores between the first and second renal biopsies of > 1.1) | 24 | Median (IQR)  34 (20, 45) (Progressive)  34 (24, 38) (Stable) | 7/17 | Median (IQR) 4.3 years (1, 6) |
|  |  |  | 2. Stable (ratio ≤ 1.1; hyperplasia ≤ 10%) |  |  |  |  |
| Liu D^25^ 2019^a^ | Retrospective cohort study | Renal endpoint | 1. ESRD | 455 | Mean±SD 32.03±11.87 | 253/202 | Median 42.2 months |
|  |  |  | 2. An irreversible 50% eGFR reduction. |  |  |  |  |
| Liu J^26^ 2017 | Retrospective cohort study | Progression to ESRD | 1. ESRD was defined as GFR < 15 ml/min per 1.73 m^2^ | 349 | Mean±SD 35.42±9.53 | 160/189 | Median 84 and 76.8 months |
|  |  |  | 2. A decrease in eGFR > 50% |  |  |  |  |
| Liu LL^27^ 2018^a^ | Retrospective cohort study | Composite event | 1. A ≥50% reduction in eGFR, ESRD (if eGFR was less than 15 ml/min/1.73 m^2^ or renal replacement therapy, including haemodialysis, peritoneal dialysis or renal transplantation, was initiated) | 869 | Median 34 (range 14-77) | 428/441 | Mean±SD 44±20  months |
|  |  |  | 2. Death |  |  |  |  |
| Liu Y^28^ 2021 | Retrospective cohort study | Renal endpoint | 1. Double with initial Scr | 246 | Mean±SD 37.76±12.05 | 137/109 | six months |
|  |  |  | 2. A 50% decline in renal function during follow-up |  |  |  |  |
|  |  |  | 3. A combined renal event of ESRD (eGFR < 15 ml/min per 1.73 m^2^). |  |  |  |  |
| Ma F^29^ 2020^a^ | Retrospective cohort study | Renal function  decline and the renal survival | 1. A ≥50% reduction in eGFR | 338 | Mean±SD 32.1±11.1 | 136/202 | Mean±SD 49.9±26 months |
|  |  |  | 2. A ≥50% reduction in ESRD. |  |  |  |  |
| Mohd R^30^ 2021^a^ | Retrospective cohort study | Combined event | 1. 50% decrease in the eGFR (compared to baseline at renal biopsy) | 130 | Mean±SD 38.0±14.0 | 74/56 | Median (IQR) 7.5 years (4, 13) |
|  |  |  | 2. 50% decrease in the ESRD |  |  |  |  |
| Moriyama T^31^ 2012 | Retrospective cohort study | Progression to ESRD | 1. ESRD (required hemodialysis or renal transplantation) | 42 | Mean±SD 34.2±12.6 | 21/21 | NA |
| Moriyama T^32^ 2015 | Retrospective cohort study | Progression to ESRD | 1. ESRD (defined as requiring dialysis or renal transplantation) | 45 | NA | 18/27 | NA |
| Ouyang Y^33^ 2016 | Retrospective cohort study | The occurrence of ESRD | 1. ESRD was defined as eGFR<15 ml/min/1.73m^2^ with the need for renal replacement therapy (dialysis or renal transplantation) | 930 | Mean±SD 37.57±11.83 | 472/458 | Median 47.1 Months (range 6-246) |
| Pan M^34^ 2018 | Retrospective cohort study | Poor renal outcomes | 1. A > 50% decrease in the eGFR from the baseline level | 712 | Mean±SD  37±12.1 | 409/303 | Mean±SD 40±12.3 months |
|  |  |  | 2. Progression to ESRD |  |  |  |  |
| Pană N^35^ 2024 | Retrospective cohort study | The incidence of ESKD | 1. ESKD (initiate dialysis or undergo a kidney transplantation) | 196 | Median (IQR)  43 (41, 46) | 61/135 | 113.0 months (95% CI 108.5-117.4) |
|  |  |  | 2. The secondary endpoint was all-cause mortality |  |  |  |  |
| Park GY^36^ 2015 | Retrospective cohort study | Renal outcome | 1. CKD stage 3 or above was defined as an adverse | 91 | Mean±SD  35±13 (range 18-77) | 43/48 | Mean±SD 37.6±19.9 months |

**Table S2 (Continued)**

| **Author/ Year** | **Study design**  **(Quality score)** | **Primary outcome** | **Description of the outcome events** | **General demographic characteristics** | | | |
| --- | --- | --- | --- | --- | --- | --- | --- |
|  |  |  |  | ***n*** | **Average age (years)^a^** | **F/M ratio** | **Follow-up periods** |
| Peters HP^37^ 2011 | Retrospective cohort study | ESRD | 1. Initiation of dialysis, renal transplantation | 65 | Mean±SD 43±13 | 18/47 | Median 75 Months (range 3-146) |
|  |  |  | 2. An eGFR <15 mL/min/1.73m^2^ |  |  |  |  |
| Qi C^38^ 2025 | Retrospective cohort study | Doubling of creatinine or ESRD | 1. Maintenance hemodialysis, peritoneal dialysis and kidney transplantation | 718 | Mean±SD 36.32±11.10 | 353/365 | Median 52.63 months |
| Rhee H^39^ 2015^a^ | Retrospective cohort study | Renal progression | 1. An eGFR decline > 50% from baseline | 121 | Mean±SD 32.74±13.16 | 49/72 | Median 41.49 months |
|  |  |  | 2. Progression to ESRD |  |  |  |  |
| Saleem N^40^ 2024 | Retrospective cohort study | Renal outcome | 1. A ≥50% decline in eGFR from the time of renal biopsy | 93 | Median (IQR)  29 (10, 80) | 30/63 | Median (IQR) 12 months (6, 67) |
|  |  |  | 2. The development of ESRD |  |  |  |  |
| Shin DH^41^ 2016 | Retrospective cohort study | The onset of ESRD and 50% reduction in eGFR | 1. ESRD (Initiation of renal replacement therapy, including  permanent hemodialysis, peritoneal dialysis, or renal transplantation) | 627 | Median (IQR)  32 (25, 42) | 325/302 | Median (IQR) 48.0 months (28.7, 76.7) |
|  |  |  | 2. 50% reduction in eGFR |  |  |  |  |
| Tan J^42^ 2022 | Retrospective cohort study | ESRD | 1. An eGFR less than 15 mL/min/1.73 m^2^ and/or | 966 | Mean±SD 34.52±11.30 | 448/518 | Mean±SD  58.67±28.53 |
|  |  |  | 2. The start of renal replacement therapy |  |  |  |  |
| Tan L^43^ 2021^a^ | Observational study | Primary endpoint | 1. A 50% decline of eGFR | 1239 | Mean±SD 34.82±11.03  33.23±11.25  31.71±10.21 | 702/537 | Mean±SD 48.90±23.86 |
|  |  |  | 2. ESRD (ESRD was defined as eGFR < 15 mL/min/1.73 m^2^  or maintenance renal replacement treatment) |  |  |  |  |
|  |  |  | 3. Death |  |  |  |  |
| Tang T^44^ 2025 | Retrospective cohort study | Combined event | 1. A≥50% decrease in the eGFR from the baseline level | 356 | Mean±SD 37.58±11.21 | 207/149 | Mean±SD 4.65±0.93 years |
|  |  |  | 2. The incidence of ESKD |  |  |  |  |
| Tian ZY^45^ 2023 | Retrospective cohort study | Renal outcome | 1. The progression to ESRD (defined by commencement of renal replacement therapy) | 519 | Median (IQR) 32 (25,42) | 266/253 | Median (IQR) 61 months (31,89) |
|  |  |  | 2. An eGFR<15 ml·min-1· (1.73 m^2^)-1 |  |  |  |  |
| Torres DD^46^ 2008 | Prospective cohort study | Outcome measures | 1. Doubling of baseline serum creatinine (sCr) and/or | 132 | Mean±SD 31.6±11.4 | 41/91 | Median (IQR) 54 months (35, 84） |
|  |  |  | 2. ESRD (defined as the need for regular dialysis treatment or renal transplant) |  |  |  |  |
| Walsh M^47^ 2010^a^ | Prospective cohort study | Composite end point | 1. Doubling of baseline serum creatinine | 146 | Mean±SD  38±13 | 51/95 | Median 5.8 years |
|  |  |  | 2. ESRD (permanent hemodialysis, peritoneal dialysis, or renal transplantation) |  |  |  |  |
|  |  |  | 3. Death from any cause |  |  |  |  |
| Wang S^48^ 2021 | Prospective cohort study | ESRD | 1. eGFR of <15 mL/min/1.73 m^2^ | 966 | Median (IQR) 35 (26, 42) | 523/443 | Median 58.67 months |
|  |  |  | 2. Performance of renal replacement therapy |  |  |  |  |
| Wang Y^49^ 2024 | Prospective cohort study | Composite of ESRD | 1. ESRD was defined as eGFR < 15 ml/min/1.73m^2^ | 69 | Median (IQR) 43.0 (31.0, 50.0) | 36/33 | Median (IQR) 18.0 months (12.0, 36.0) |
|  |  |  | 2. The initiation of maintenance dialysis or renal transplantation |  |  |  |  |
| Wang Ying^50^ 2024 | Prospective cohort study | Composite of ESRD and death | 1. ESRD was defined as eGFR < 15 ml/min/1.73 m^2^ | 75 | Median (IQR) 42.0 (31.0, 50.0) | 37/38 | Median 15 months |
|  |  |  | 2. The initiation of maintenance dialysis or renal transplantation |  |  |  |  |

**Table S2 (Continued)**

| **Author/ Year** | **Study design**  **(Quality score)** | **Primary outcome** | **Description of the outcome events** | **General demographic characteristics** | | | |
| --- | --- | --- | --- | --- | --- | --- | --- |
|  |  |  |  | ***n*** | **Average age (years)^a^** | **F/M ratio** | **Follow-up periods** |
| Worawichawong S^51^ 2021 | Retrospective cohort study | Combined adverse kidney outcome | 1. Kidney Failure (the need for kidney replacement therapy or eGFR <15 ml/min/1.73m^2^) | 120 | Mean±SD 37.3±12.6 | 64/56 | Mean±SD  51.6±17.1 months |
|  |  |  | 2. A decline of eGFR by more than 50% |  |  |  |  |
| Wu D^52^ 2021 | Retrospective cohort study | Composite kidney outcome | 1. A 30% decline in eGFR from the baseline | 98 | Median (IQR) 9.7 (6.6, 12.2) | 32/66 | Median (IQR) 25 months (  18, 36) |
|  |  |  | 2. Kidney failure (initiated kidney replacement therapy, including dialysis or kidney transplantation) |  |  |  |  |
| Xia M^53^ 2020^a^ | Retrospective cohort study | Renal outcome | 1. An ESRD | 291 | Mean±SD 32.50±11.94 | 160/131 | Median 41.2 months |
|  |  |  | 2. An irreversible 50% eGFR reduction |  |  |  |  |
| Xie J^54^ 2018 | Retrospective cohort study | ESRD | 1. First dialysis or kidney transplantation | 934 | Mean±SD 36.5±12.0 | 472/462 | Median (IQR) 56.3 months (12.0, 300.1) |
|  |  |  |  | 1221 | Mean±SD 36.1±11.7 | 619/602 | Median (IQR) 47.8 months (12, 204) |
| Xie J^55^ 2012 | Retrospective cohort study | Occurrence of ESRD | 1. ESRD is defined by a need for renal replacement therapy (dialysis or renal transplantation) | 619 | Mean±SD 36.0±12.3 | 305/314 | Mean (scope), 41.3 months (3.03–248.1) |
|  |  |  | 2. A 50% decline from baseline eGFR |  |  |  |  |
| Xing Y^56^ 2024^a^ | Retrospective cohort study | The composite kidney endpoint events | 1. A doubling of the baseline serum creatinine, 40% reduction in eGFR, ESRD (eGFR <15 ml/min per 1.73 m^2^), dialysis, transplant, or death. | 1094 | Mean±SD 38.5±12.7 | 581/513 | NA |
|  |  |  |  | 113 | Mean±SD 37.1±12.2 | 64/49 | NA |
| Xu X^57^ 2022^a^ | Retrospective cohort study | Composite event | 1. A reduction in eGFR of≥50% | 105 | Median (IQR) 36 (28, 43) | 54/51 | Median (IQR) 37 months (27.5, 59.0) |
|  |  |  | 2. ESRD |  |  |  |  |
|  |  |  | 3. Death |  |  |  |  |
| Yang WG^58^ 2023 | Retrospective cohort study | Composite event | 1. A ≥ 50% reduction in eGFR | 935 | Mean±SD   37.96±12.71 | 447/488 | Mean±SD  47±15 months |
|  |  |  | 2. ESRD (eGFR < 15 ml/min/1.73 m^2^, dialysis, or renal transplantation) |  |  |  |  |
| Yang Y^59^ 2020^a^ | Retrospective cohort study | Composite endpoint | 1. eGFR decline >50% | 642 | Median (IQR) 33.23 (26.83, 42.50) | 361/281 | Median 43.18 months |
|  |  |  | 2. Doubling of baseline serum creatinine |  |  |  |  |
|  |  |  | 3. ESRD (eGFR <15 ml/min/1.73 m^2^, dialysis, and kidney transplantation) |  |  |  |  |
| Yoon SY^60^ 2024 | Retrospective cohort study | CKD progression | 1. A > 20% decrease in eGFR from baseline levels | 191 | Mean±SD  41.3±15.3 | 92/99 | One year after the biopsy |
|  |  |  | 2. Renal replacement therapy |  |  |  |  |
|  |  |  | 3. Kidney transplantation |  |  |  |  |
| Yu G^61^ 2021^a^ | Retrospective cohort study | Composite endpoint | 1. A 50% eGFR decline | 1818 | Mean±SD  38.61±12.20 | 862/956 | Median (IQR) 25.67 months (13.03, 47.44) |
|  |  |  | 2. ESKD (was defined as eGFR < 15 mL/min/1.73 m^2^ or the need for kidney replacement therapy, including hemodialysis, peritoneal dialysis, or kidney transplantation). |  |  |  |  |
|  |  |  | 3. Death (whichever occurred first) |  |  |  |  |

**Table S2 (Continued)**

| **Author/ Year** | **Study design**  **(Quality score)** | **Primary outcome** | **Description of the outcome events** | **General demographic characteristics** | | | |
| --- | --- | --- | --- | --- | --- | --- | --- |
|  |  |  |  | ***n*** | **Average age (years)^a^** | **F/M ratio** | **Follow-up periods** |
| Yu Z^62^ 2023^a^ | Retrospective cohort study | Renal endpoint | 1. Progression to ESRD | 263 | Median (IQR) 36 (29, 47) | 83/180 | Median (IQR) 57.32 months (32.05, 82.96) |
|  |  |  | 2. Death |  |  |  |  |
| Zagorec N^63^ 2024 | Retrospective cohort study | Kidney failure | 1. A permanent decline in eGFR < 15 mL/min/1.73 m^2^ | 95 | Median (IQR) 44.6 (32.0, 52.2) | 27/68 | Median (IQR) 102.2 months (72.1, 138.2) |
|  |  |  | 2. KRT (dialysis or kidney transplantation, commencement) |  |  |  |  |
| Zhai Y^64^ 2024 | Retrospective cohort study | ESKD | 1. An eGFR <15 mL/min/1.73 m^2^ | 374 | Median (IQR) 33 (25, 44) | 177/197 | Median 32.5 months |
|  |  |  | 2. The initiation of renal replacement therapy |  |  |  |  |
| Zhang J^65^ 2017 | Prospective cohort study | Poor renal outcomes | 1. A 50% decrease in the eGFR | 672 | Median 36 (range 28-45) | 383/289 | Median 36 months (range 19-61) |
|  |  |  | 2. ESRD |  |  |  |  |
| Zhao YF^66^ 2016 | Prospective cohort study | Composite end point | 1. 30% eGFR decline | 438 | Mean±SD  35.6±12.4 | 200/238 | Median (IQR) 37.0 months (22.0, 58.0) |
|  |  |  | 2. ESRD (eGFR<15 ml/min per 1.73 m^2^ or need for KRT, including hemodialysis, peritoneal dialysis, or renal transplantation) |  |  |  |  |
|  |  |  | 3. Death (whichever occurred first) |  |  |  |  |

Abbreviations: ESRD, end-stage renal disease; F/M, female/male; eGFR, estimated glomerular filtration rate; IQR, interquartile range; Ccr, creatinine clearance; CKD, chronic kidney disease; KRT, kidney replacement therapy; ESKD, end-stage kidney disease; NA, not available; SD, standard deviation.

a: The studies were categorized into severity subgroups.
